# Abrogation of graft ischemia‐reperfusion injury in ischemia‐free liver transplantation

**DOI:** 10.1002/ctm2.546

**Published:** 2022-04-26

**Authors:** Zhiyong Guo, Jinghong Xu, Shanzhou Huang, Meixian Yin, Qiang Zhao, Weiqiang Ju, Dongping Wang, Ningxin Gao, Changjun Huang, Lu Yang, Maogen Chen, Zhiheng Zhang, Zebin Zhu, Linhe Wang, Caihui Zhu, Yixi Zhang, Yunhua Tang, Haitian Chen, Kunpeng Liu, Yuting Lu, Yi Ma, Anbin Hu, Yinghua Chen, Xiaofeng Zhu, Xiaoshun He

**Affiliations:** ^1^ Organ Transplant Centre, The First Affiliated Hospital Sun Yat‐sen University Guangzhou China; ^2^ Guangdong Provincial Key Laboratory of Organ Donation and Transplant Immunology Guangzhou China; ^3^ Guangdong Provincial International Cooperation Base of Science and Technology (Organ Transplantation) Guangzhou China; ^4^ Department of Anaesthesiology, The First Affiliated Hospital Sun Yat‐sen University Guangzhou China

**Keywords:** ischemia‐free liver transplantation, ischemia‐reperfusion injury, metabolomics, transcriptomics

## Abstract

**Background:**

Ischemia‐reperfusion injury (IRI) is considered an inherent component of organ transplantation that compromises transplant outcomes and organ availability. The ischemia‐free liver transplantation (IFLT) procedure has been developed to avoid interruption of blood supply to liver grafts. It is unknown how IFLT might change the characteristics of graft IRI.

**Methods:**

Serum and liver biopsy samples were collected from IFLT and conventional liver transplantation (CLT) recipients. Pathological, metabolomics, transcriptomics, and proteomics analyses were performed to identify the characteristic changes in graft IRI in IFLT.

**Results:**

Peak aspartate aminotransferase (539.59 ± 661.76 U/L versus 2622.28 ± 3291.57 U/L) and alanine aminotransferase (297.64 ± 549.50 U/L versus 1184.16 ± 1502.76 U/L) levels within the first 7 days and total bilirubin levels by day 7 (3.27 ± 2.82 mg/dl versus 8.33 ± 8.76 mg/dl) were lower in the IFLT versus CLT group (all *p* values < 0.001). The pathological characteristics of IRI were more obvious in CLT grafts. The antioxidant pentose phosphate pathway remained active throughout the procedure in IFLT grafts and was suppressed during preservation and overactivated postrevascularization in CLT grafts. Gene transcriptional reprogramming was almost absent during IFLT but was profound during CLT. Proteomics analysis showed that “metabolism of RNA” was the major differentially expressed process between the two groups. Several proinflammatory pathways were not activated post‐IFLT as they were post‐CLT. The activities of natural killer cells, macrophages, and neutrophils were lower in IFLT grafts than in CLT grafts. The serum levels of 14 cytokines were increased in CLT versus IFLT recipients.

**Conclusions:**

IFLT can largely avoid the biological consequences of graft IRI, thus has the potential to improve transplant outcome while increasing organ utilization.

## Graphical abstract

Ischemia free liver transplantation (IFLT) procedure can largely avoid the biological consequences of graft ischemia‐reperfusion injury.

IFLT maintains active pentose phosphate pathway (PPP) metabolism and redox homeostasis.

IFLT prevents hepatocytes death and the activation of inflammation and immune response.

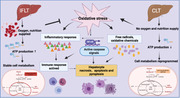

**Abbreviations**
ADP: adenosine diphosphate
ALT: alanine aminotransferase
AST: aspartate aminotransferase
ATP: adenosine triphosphate
CLT: conventional liver transplantation
COTRS: China Organ Transplant Response System
CXCL1: C‐X‐C motif ligand‐1
DBD: donation after brain death
DEG: differentially expressed gene
ECD: extended criteria donor
ELISA: enzyme‐linked immunosorbent assay
EP: end of preservation
G6PDH: glucose‐6‐phosphate dehydrogenase
GEO: Gene Expression Omnibus
GM‐CSF: granulocyte‐macrophage colony stimulating factor
GSDMD: gasdermin D
GSH: glutathione
HE: hematoxylin and eosin
IFLT: ischemia‐free liver transplantation
IRI: ischemia‐reperfusion injury
KEGG: Kyoto Encyclopedia of Genes and Genomes
LC‐MS/MS: liquid chromatography‐tandem mass spectrometry
MAPK: mitogen‐activated protein kinase
MCP: monocyte chemoattractant protein
MDA: malondialdehyde
NF‐κB: nuclear factor kappa B
NK: nature killer
NLRP3: NOD‐like receptor protein‐3
NMP: normothermic machine perfusion
OPLS‐DA: orthogonal partial least squares‐discriminant analysis
PCA: principal components analysis
PERMANOVA: permutational multivariate analysis of variance
PP: preprocurement
PPP: pentose phosphate pathway
PR: one‐hour postgraft revascularization
ROS: reactive oxygen species
RT‐qPCR: quantitative reverse transcription polymerase chain reaction
SCD: standard criteria donor
SD: standard deviation
SOD: superoxide dismutase
TBIL: total bilirubin
TEM: transmission electron microscopy
TNF: tumor necrosis factor
TUNEL: TdT‐mediated dUTP nick end labeling
VIP: variable importance plot
vWF: von Willebrand factor

## 1 BACKGROUND

Ischemia and reperfusion injury (IRI) is an inherent component of liver transplantation, resulting in severe complications such as primary nonfunction, ischemic‐type biliary lesions, and postreperfusion syndrome.^1^ To overcome the increasing organ shortage, extended criteria donor (ECD) organs are frequently used to expand the donor pool. However, compared to standard criteria donor (SCD) organs, those organs are more sensitive to IRI and have higher risks of graft loss.^2^

A number of methods have been proposed to reduce IRI, such as ischemic preconditioning, pharmacological or gene interventions, and stem cell therapy.^3^ Recently, the efficacy and safety of various types of machine perfusion technologies for organ preservation have been tested, such as hypothermic (oxygenated) machine perfusion, subnormothermic machine perfusion, normothermic machine perfusion (NMP), and controlled oxygenated rewarming perfusion.^4^, ^5^, ^6^, ^7^, ^8^, ^9^, ^10^, ^11^ Nevertheless, because grafts still suffer ischemia during procurement and implantation, those approaches are unable to completely abrogate the subsequent IRI.

To avoid graft ischemia entirely, we introduced an ischemia‐free liver transplantation (IFLT) procedure. The procedure is designed to procure, preserve, and implant liver grafts under continuous NMP.^12^ In this study, we aimed to test how this procedure could change the biological consequences of graft IRI by analyzing serum and liver biopsy samples collected from the first nonrandomized controlled study.

## 2 METHODS

### 2.1 Study design

**FIGURE 1:**
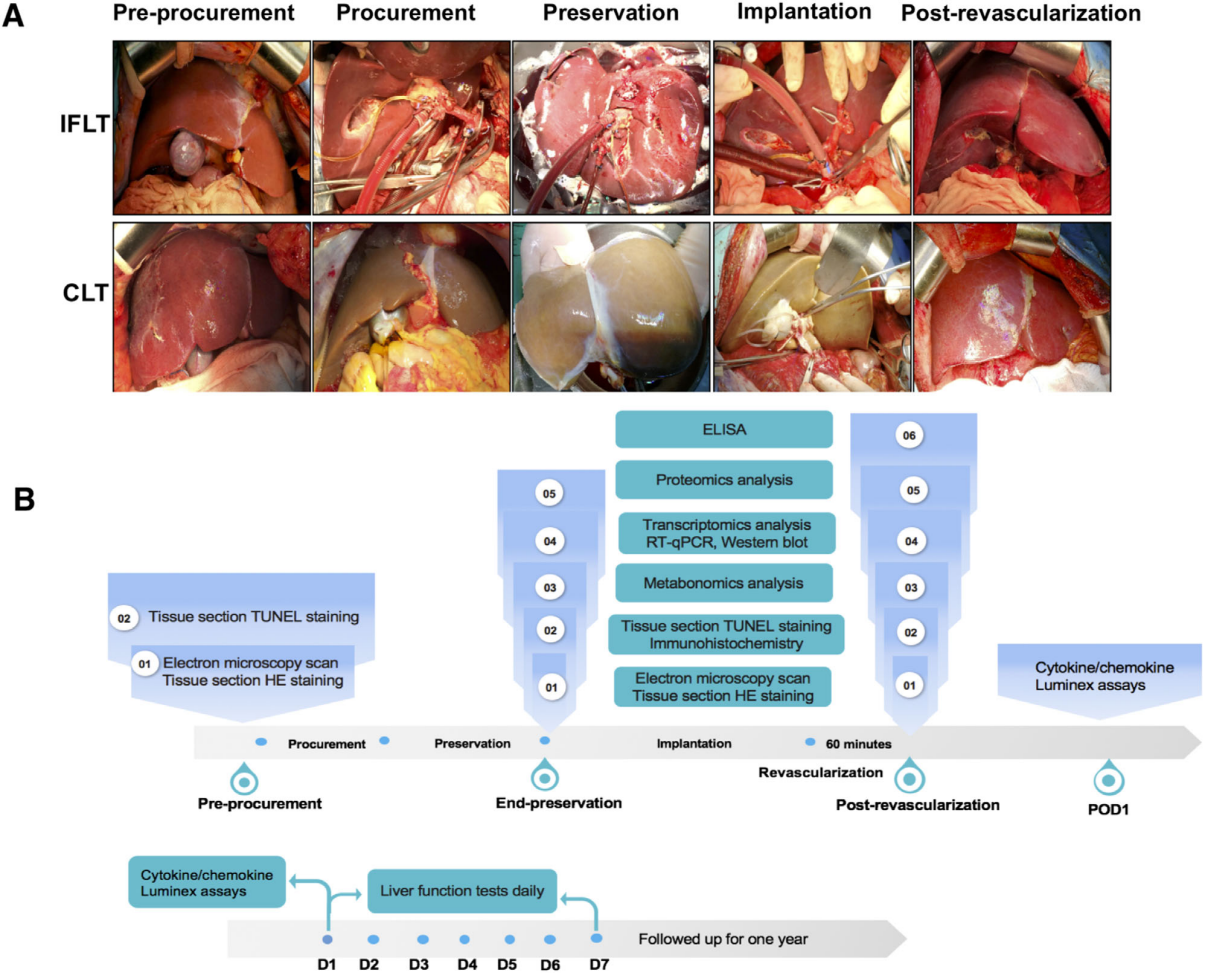
Technical comparison of IFLT versus CLT and the flow chart of the study design. (A) The main steps of the two liver transplant procedures. (B) Biological analysis of liver biopsy samples during the operation (top) and blood samples after the operation (bottom). Abbreviations: CLT, conventional liver transplantation; CS, cold store; NMP, normothermic machine perfusion; HE, hematoxylin and eosin; IFLT, ischemia‐free liver transplantation; RT‐qPCR, quantitative reverse transcription polymerase chain reaction; TUNEL, terminal‐deoxynucleotidyl transferase‐mediated nick end labeling

In our single‐center, nonrandomized, controlled trial (ChiCTR‐OPN‐17012090), the efficacy, and safety were compared between IFLT and conventional liver transplantation (CLT). Liver biopsy samples were collected preprocurement (PP), at the end of preservation (EP) and one‐hour postgraft revascularization (PR), and serum samples were collected postoperatively to evaluate the severity of graft IRI (Figure 1).

### 2.2 Liver function tests

Blood samples were collected at 7 am each day for aspartate aminotransferase (AST), alanine aminotransferase (ALT), and total bilirubin (TBIL) measurement within 7 days posttransplantation. The detection was performed in clinical biochemistry laboratory of The First Affiliated Hospital of Sun Yat‐sen University with Automatic Chemical Analyzer 7600‐100 (Hitachi, Ltd, Tokyo, Japan).

### 2.3 Histological analysis

All liver samples were stored in 10% buffered formalin, immersed into paraffin, sliced (5 mm), and stained with hematoxylin and eosin (HE). Liver IRI was evaluated in a blinded manner by two pathologists by calculating the Suzuki score.^13^ When the scores were different, the pathologists engaged in a discussion and reached a final agreement. Samples were obtained from the first 14 IFLT cases and 14 randomly selected CLT cases.

### 2.4 Transmission electron microscopy

Liver tissues were soaked in 2.5% glutaraldehyde in 0.1 M phosphate buffer (pH = 7.2) for over 4 h at 4°C, dehydrated in graded solutions of ethanol for 10 min each time, and then infiltrated and embedded in resin to prepare ultrathin 70‐nm sections. The sections were assessed using Transmission electron microscopy (TEM) (FEI Tecnai G2 Spirit Twin, Hillsboro, USA).

### 2.5 TdT‐mediated dUTP nick end labeling assay

TdT‐mediated dUTP nick end labeling (TUNEL) assays was conducted in accordance with the manufacturer's (Roche, 11684817910, USA) instructions to detected apoptosis. Samples were obtained from the first 14 IFLT cases and 14 randomly selected CLT cases. Apoptosis was quantified by TUNEL‐positive cell number (mean ±SD), and Student's *t*‐test was used for statistical analysis.

### 2.6 Superoxide dismutase and malondialdehyde levels

A portion of the liver tissue was homogenized in saline solution to analyze superoxide dismutase (SOD) and malondialdehyde (MDA) levels. The levels in samples were quantified with the relevant detection kits in accordance with the manufacturer's (Nanjing Jian‐Cheng Bioengineering Institute, Nanjing, China) instructions.

### 2.7 Quantitative reverse transcription‐polymerase chain reaction (RT‐qPCR)

Total RNA extraction and cDNA synthesis were conducted as previously described,^14^ and quantitative reverse transcription‐polymerase chain reaction (RT‐qPCR) was conducted as the instruction of the SYBR Green Quantitative PCR kit (Takara Bio, Inc.). The related primer sequences are shown in Table S1. Relative mRNA expression was expressed by the 2^–△△CT^, and Student's *t*‐test was used to evaluate significant differences.

### 2.8 Immunohistochemistry

Liver tissue paraffin sections were deparaffinized, performed for antigen retrieval, blocked endogenous peroxidases activity with 3% H_2_O_2_, and blocked with serum. The primary antibody was incubated for 2 h at 37°C. The secondary antibody was incubated for 30 min at room temperature. Following diaminobenzidine coloration, the slides were stained with hematoxylin for 3 min, costained with hematoxylin and coverslipped. Antibodies against von Willebrand factor (vWF) (Servicebio, GB11020) were purchased and used. Staining was assessed by pathologists who were blinded to the sample origins.

### 2.9 Immunoblot analysis

Tissue proteins harvested RIPA buffer (Fisher, Rockford, IL, USA) were prepared for separation in SDS‐PAGE gel and then transferred to PVDF membranes (Bio‐Rad). The membranes were blocked with 5% skim milk and incubated in the appropriate primary antibody, followed by incubation with a secondary antibody. The target protein bands were detected by a protein imaging system, Image Lab (ChemiDoc XRS+, BioRad, USA).

### 2.10 Enzyme‐linked immunosorbent assay (ELISA)

The preparation of tissue supernatant and the detection of interleukin (IL)‐1β and IL‐18 was performed according to the instruction manual of enzyme‐linked immunosorbent assay (ELISA) kits (Enzyme‐linked Biotechnology Co., Shanghai, China). The absorbance of the samples was measured at 450 nm using a microplate reader, and the concentration of IL‐1β and IL‐18 was calculated according to the standard curve formula.

### 2.11 Metabolomics analysis

Liver biopsy samples were collected from the first 20 IFLT cases and 20 randomly selected CLT cases. The supernatant was prepared from 20 mg of liver tissue. A UHPLC system (1290, Agilent Technologies) was used to couple UPLC BEH Amide column (1.7 μm 2.1*100 mm, Waters) to TripleTOF 6600 (Q‐TOF, AB Sciex and QTOF 6550 (Agilent) was performed by liquid chromatography‐tandem mass spectrometry (LC‐MS/MS). The LC/MS assay method and the database used for analysis were described in a previously published article.^15^ The in‐house MS2 database was used for metabolite identification. The intrinsic variations of the data were illustrated by principal component analysis (PCA) and orthogonal projections to latent structure‐discriminant analysis (OPLS‐DA) score plots. Permutational multivariate analysis of variance (PERMANOVA) was used to assess differences in metabolite changes between groups. Variable importance plots (VIP) > 1 and *p *< 0.05 were used to distinguish significantly differentially abundant metabolites. The screening results of differential metabolites were visualized using a volcano plot. The enrichment analysis and topological analysis of the pathways were used to identify the key pathways related to the differentially expressed metabolites. The bubble plot shows the results of the metabolic pathway analysis.

### 2.12 Transcriptome analysis

Liver biopsy samples were obtained from the first 14 IFLT cases and 14 randomly selected CLT cases. The analysis was based on our previous publication.^15^ Genes greater than two‐fold change and *P*_adj_ < 0.01 between two groups were considered as differentially expressed. The raw data were uploaded to the NCBI Gene Expression Omnibus (GEO, accession number GSE113024).

### 2.13 Proteomics analysis

Protein was extracted from liver biopsies from 12 IFLT cases and 12 CLT cases at EP and PR. Protein from each sample was digested into peptides using MS‐grade Tyrisin (Promega, Madison, WI). The peptides were resuspended in 0.1% formic acid aqueous solution, and iRT peptide was added. The mixed liquor was tested by on‐line nanospray LC‐MS/MS on an Orbitrap Fusion Lumos mass spectrometer (Thermo Fisher Scientific, MA, USA) coupled to an EASY‐nanoLC 1200 system (Thermo Fisher Scientific). Direct data independent acquisition (DIA) analysis was performed on DIA data using Spectronaut 14 (Biognosys AG, Switzerland). The expression levels of proteins with greater than 1.5‐ or less than ‐1.5‐fold changes and *P*_adj_ < 0.05 were considered as differentially expressed. The Metascape and MetaCore online databases were used for enrichment analysis of differential proteins.

### 2.14 Cytokine/chemokine Luminex assay

Peripheral venous blood samples from 11 IFLT recipients and 14 CLT recipients were collected using EDTA as an anticoagulant at 12 h after graft revascularization. The plasma samples obtained by centrifugation for 10 min at 1000×g were stored at ‐20°C and completely dissolved before use. The assay of cytokine/chemokine measurement was directed by the manufacturer's instructions of Human 38‐plex magnetic cytokine/chemokine kits (EMD Millipore, HCYTMAG‐60K‐PX38). Fluorescence was quantified using a Luminex 200 instrument, and the concentrations were calculated by Milliplex Analyst software version 4.2 (EMD Millipore). All samples were examined in triplicate.

## 3 RESULTS

### 3.1 Clinical outcomes posttransplantation

During the study period, 38 IFLT recipients and 130 CLT recipients were recruited. The detailed clinical data are described in our recently published paper.^16^ There was no significant difference in donor and recipient characteristics between the two groups. IFLT recipients had a lower incidence of early allograft dysfunction, and higher one‐year graft/patient rates than CLT recipients.

### 3.2 Posttransplant liver function tests results

**FIGURE 2:**
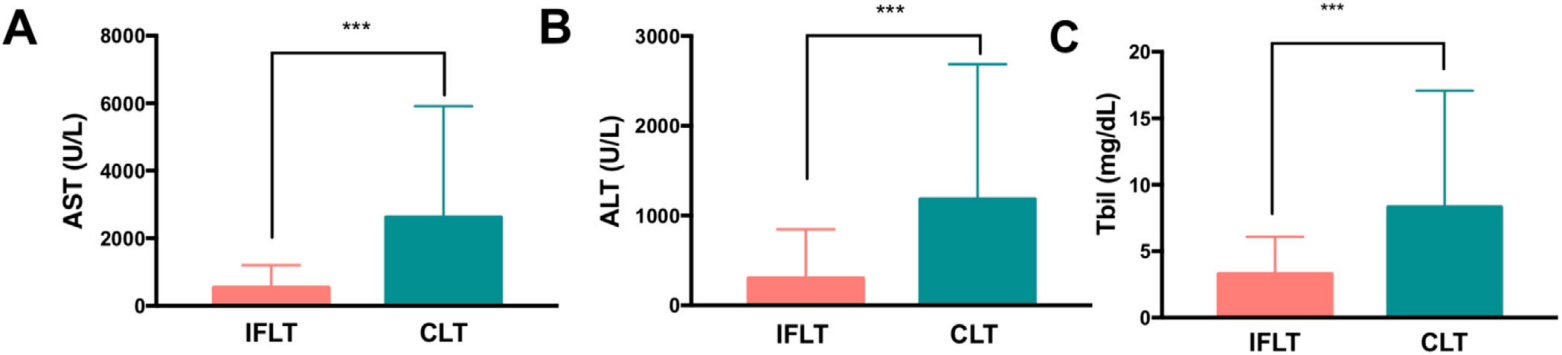
IFLT ameliorated liver IRI. Peak levels of AST (A) and ALT (B) within 7 days and TBIL levels (C) on day 7 in IFLT and CLT recipients. Abbreviations: ALT, alanine aminotransferase; AST, aspartate aminotransferase; CLT, conventional liver transplantation; IFLT, ischemia‐free liver transplantation; IRI, ischemia‐reperfusion injury; TBIL, total bilirubin

Patients undergoing IFLT had lower peak AST levels (539.59 ± 661.76 U/L vs. 2622.28 ± 3291.57 U/L, *p *< 0.001) and ALT levels (297.64 ± 549.50 U/L vs. 1184.16 ± 1502.76 U/L, *p *< 0.001) than those undergoing CLT within the first 7 days posttransplantation (Figure 2A, B). IFLT recipients had lower TBIL levels (3.27 ± 2.82 mg/dl vs. 8.33 ± 8.76 mg/dl, *p *< 0.001) by day 7 than CLT recipients (Figure 2C).

### 3.3 Metabolomics profiles of IFLT versus CLT grafts

**FIGURE 3:**
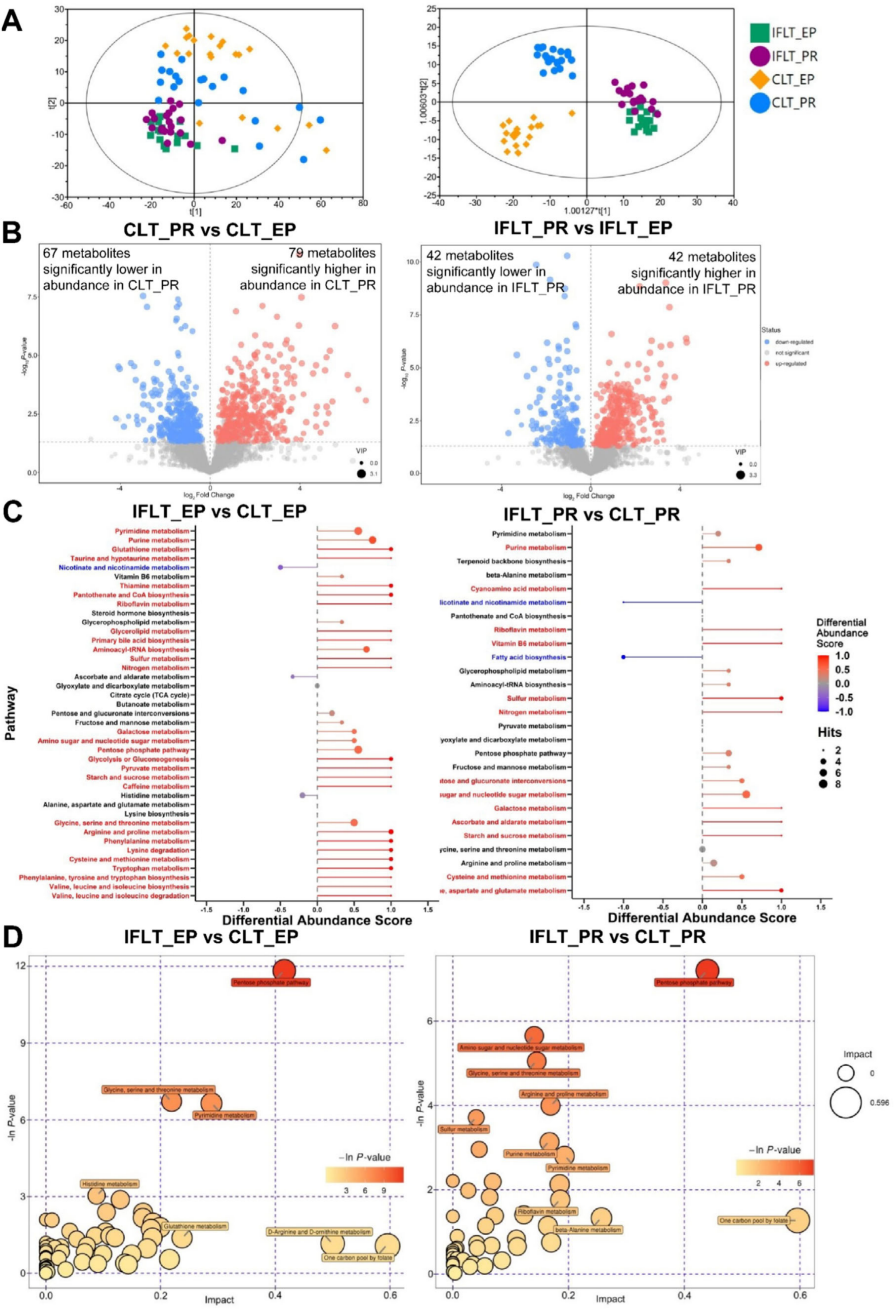
IFLT maintained active pentose phosphate pathway (PPP) metabolism and redox homeostasis. (A) PCA (left) and OPLS‐DA (right) score plots of LC‐MS/MS metabolomics data in the four subgroups. (B) Volcano plot showing CLT_PR vs CLT_EP (left) and IFLT_PR vs IFLT_EP (right). Each point in the volcano plot represents a metabolite, and the larger the scatter, the greater the VIP value. The scatter color represents the final screening result, metabolites that were significantly upregulated are red, metabolites that were significantly downregulated are blue, and metabolites that were not significantly different are gray. (C) Pathway‐based analysis of metabolic changes between IFLT_EP and CLT_EP and between IFLT_PR and CLT_PR. The differential abundance score shows the average gross changes of all metabolites in a pathway. A score of 1 indicates that all measured metabolites in the pathway increased, and −1 indicates that all measured metabolites in a pathway decreased. #, downregulated pathways; *, upregulated metabolism. (D) Pathway analysis of IFLT_EP vs CLT_EP and IFLT_PR vs CLT_PR. A larger bubble indicates a larger influence of the pathway, as shown by topological analysis. A darker color indicates more significant enrichment in the pathway Abbreviations: CLT, conventional liver transplantation; EP, end‐preservation; IFLT, ischemia‐free liver transplantation; PR, postrevascularization

During the transplant procedure, the IFLT grafts were under continuous normothermic and oxygenated perfusion. In contrast, CLT grafts were under static hypothermic and hypoxic conditions. Therefore, the metabolic activities were substantially different between the two groups, which might impact the pathogenesis of graft IRI. Therefore, the relative abundances of metabolites in the liver biopsies were measured using an LC‐MS/MS‐based nontargeted metabolomics method. It was difficult to distinguish the liver biopsy samples collected at EP from those collected PR by using both PCA and OPLS‐DA models in the IFLT group (*p *= 0.067). However, the metabolomics profiles could easily distinguish samples collected at EP and PR in the CLT group (*p = *0.028) (Figure 3A).

A volcano plot showed that among the 780 detected (146 annotated and 634 unannotated) metabolites in these samples, there were 146 differentially expressed metabolites (DEMs) (67 lower and 79 higher, VIP > 1, *p *< 0.05) between the CLT_PR and CLT_EP samples, while among the 587 detected (84 annotated and 503 unannotated) metabolites in these samples, there were 84 DEMs (42 lower and 42 higher, VIP > 1, *p *< 0.05) in IFLT_PR samples compared to IFLT_EP samples (Figure 3B, Tables S2 and S3). The abundances of a number of amino acids and derivatives (three lower, 14 higher), nucleosides, nucleotides and derivatives (18 lower, six higher), and organic acid (six lower, 17 higher) metabolites were significantly different between CLT_PR and CLT_EP samples (Table S4). Among the 41 metabolic pathways with at least two metabolites captured, 28 were higher (differential abundance score ≥ 0.5, red), and one was lower (differential abundance score ≤ ‐0.5, blue) in IFLT_EP samples than in CLT_EP samples (Figure 3C, left). Similarly, among the 27 metabolic pathways, 13 were higher, and two were lower in IFLT_PR samples than in CLT_PR samples (Figure 3C, right). Interestingly, most of the upregulated pathways in IFLT_EP relative to CLT_EP were related to amino acid, lipid, and carbohydrate metabolism, whereas the downregulated pathway was related to cofactors and vitamins metabolism. Most of the upregulated pathways in IFLT_PR relative to CLT_PR were related to nucleotide, cofactors and vitamins, energy, and carbohydrate metabolism, whereas the downregulated pathways were related to cofactors and vitamins, and lipid metabolism. Collectively, these results showed a clear metabolic reprogramming from catabolism during preservation to biosynthesis PR during CLT. In contrast, this reprogramming was not obvious during IFLT.

**FIGURE 4:**
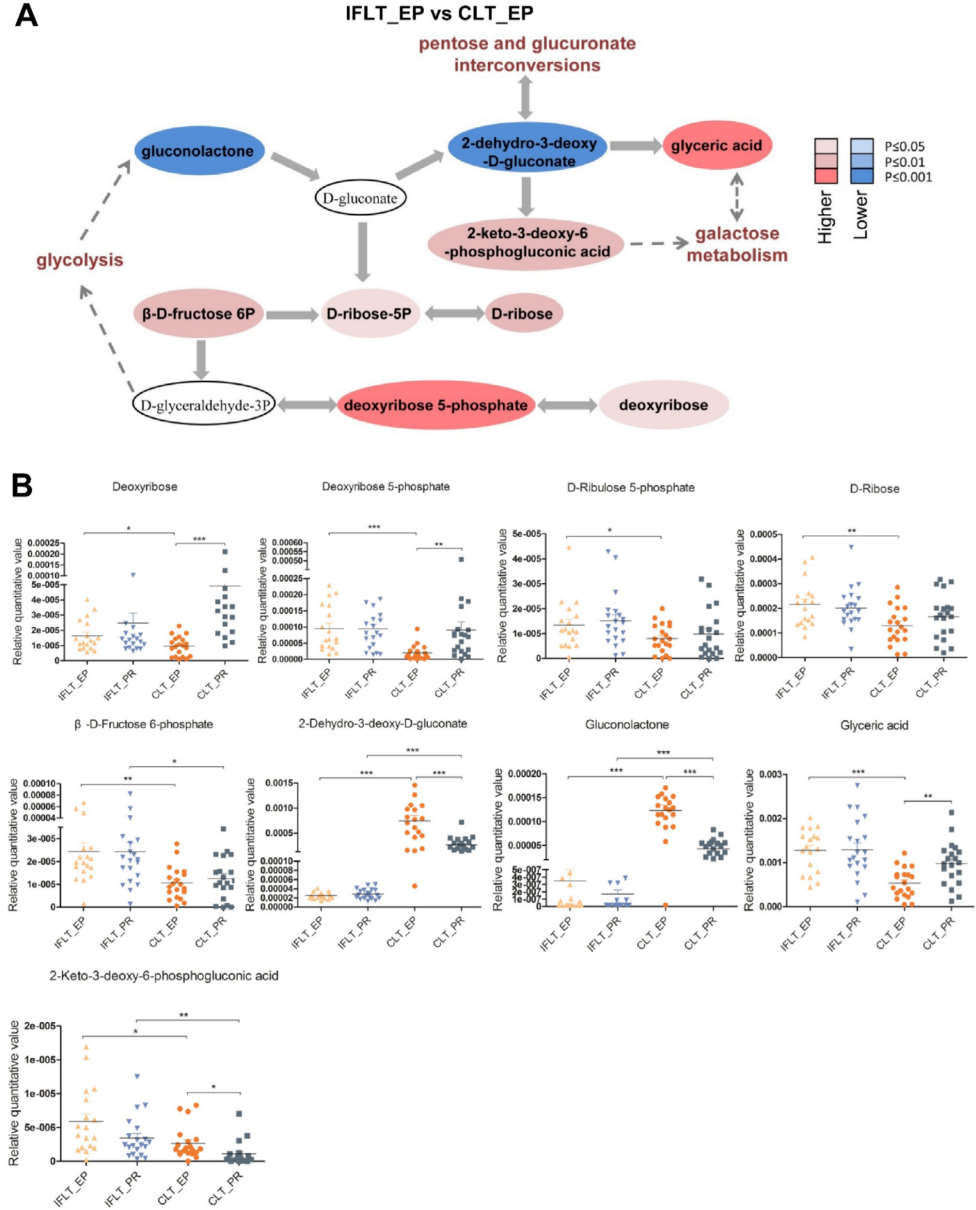
Changes in pentose phosphate pathway (PPP) metabolites in the IFLT and CLT groups. (A) Metabolic changes in the PPP. Metabolites are labeled as color‐coded ovals. The color corresponds to the *p* value between IFLT_EP and CLT_EP. Red, increased; blue, decreased. (B) Nine of the deferentially expressed metabolites (DEMs) obtained by comparing IFLT_EP and CLT_EP belong to the PPP. In the four groups, the nine different metabolites were quantitatively compared, and scatter plots were made. *p < 0.05, **p < 0.01, ***p < 0.001. Abbreviations: CLT, conventional liver transplantation; EP, end‐preservation; IFLT, ischemia‐free liver transplantation; PR, postrevascularization

The enrichment analysis and topological analysis showed that the top one differential pathway between IFLT_EP and CLT_EP was the pentose phosphate pathway (PPP) (Figure 3D, left). The top one differential pathway between IFLT_PR and CLT_PR was also PPP (Figure 3D, right). We therefore constructed a metabolic map of the PPP. Gluconolactone and 2‐dehydro‐3‐deoxy‐D‐gluconate decreased in abundance, while glyceric acid and 2‐keto‐3‐deoxy‐6‐phosphogluconic acid increased in IFLT_EP samples compared to CLT_EP samples. Both deoxyribose 5‐phosphate and deoxyribose were significantly increased in IFLT_EP samples compared to CLT_EP samples, suggesting more active biosynthesis during IFLT_EP than CLT_EP (Figure 4A). Nine DEMs in this pathway were identified when comparing IFLT_EP grafts to CLT_EP grafts. None of these metabolites changed significantly from EP to PR in IFLT grafts. In contrast, the levels of all these metabolites except D‐ribulose 5‐phosphate, D‐ribose, and β‐D‐fructose 6‐phosphate changed significantly from EP to PR in CLT grafts. Overall, the antioxidant PPP was stable throughout IFLT, while this pathway was suppressed during preservation but overactivated PR in CLT (Figure 4B).

### 3.4 IRI‐associated pathological characteristics of IFLT versus CLT grafts

**FIGURE 5:**
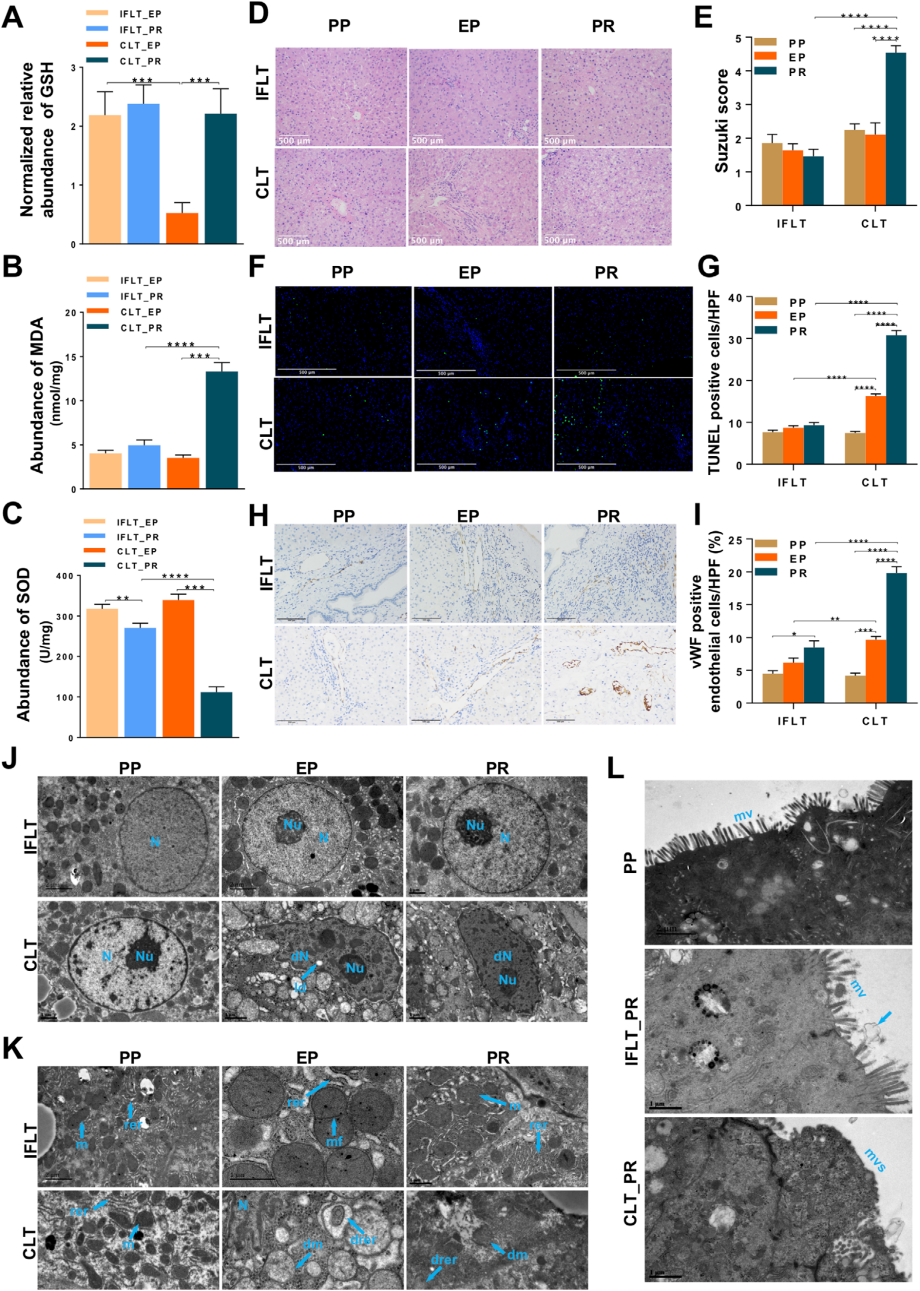
Oxidative stress and pathological changes in IFLT and CLT grafts. (A) Normalized relative abundance of glutathione (GSH). (B) The malondialdehyde (MDA) level in liver biopsies. (C) The superoxide dismutase (SOD) level in liver biopsies. (D and E) HE staining and Suzuki scoring of liver graft biopsies. (F and G) The number of apoptotic hepatocytes was measured by TUNEL assays and compared between the two groups by calculating the number of TUNEL‐positive hepatocytes per high‐power field. (H and I) Sinusoidal endothelial cell (SEC) activation was assessed by immunohistochemical staining for vWF. In total, six patients who underwent CLT and six patients who underwent IFLT were included. Samples were collected before organ procurement (PP), at the end of preservation (EP) and 1 h after graft revascularization (PR). (J and K) Transmission electron micrographs showing the nuclei (J), mitochondria and rough endoplasmic reticulum (K) in hepatocytes. (L) Transmission electron micrographs showing columnar epithelial cells in the common bile duct. arrow, bile secretion. Two‐tailed Student's t‐test was used for statistical analysis, *P < 0.05, **P < 0.01, ***P < 0.001, ****P < 0.0001. Abbreviations: CLT, conventional liver transplantation; dm, damaged mitochondria; dN, denatured nucleus; ld, lipid droplet; m. mitochondria; mf, mitochondrial proliferation; drer, damaged rough endoplasmic reticulum; IFLT, ischemia‐free liver transplantation; mv, microvilli; mvs, microvilli shedding; N, nucleus; Nu, nucleolus; rer, rough endoplasmic reticulum

In the PPP, the oxidized form of nicotinamide adenine dinucleotide phosphate (NADP^+^) is converted to nicotinamide adenine dinucleotide phosphate (NADPH). NADPH is an important reducing equivalent to replenish the reduced glutathione (GSH) pool and maintain redox homeostasis in cells.^17^ We documented stable levels of the antioxidants GSH and SOD and the oxidant MDA in liver biopsies during IFLT (Figure 5A‐C). In contrast, the MDA level was increased, while GSH and SOD levels were decreased in the CLT group. These results suggest that IFLT can maintain redox homeostasis in the grafts through the PPP.

We then assessed the histological characteristics of IRI in the liver biopsies obtained during the procedures. The Suzuki score was stable during IFLT. In contrast, a significantly increased Suzuki score was observed during CLT (Figure 5D, E). An obvious increase in the number of apoptotic hepatocytes was observed during CLT, whereas the number of apoptotic hepatocytes was stably low during IFLT (Figure 5F, G). Sinusoidal endothelial cell (SEC) activation, as shown by vWF staining, was not obvious in the IFLT group (Figure 5H, I). IFLT grafts showed almost normal ultrastructural appearances. In contrast, CLT grafts exhibited denatured nuclei, damaged mitochondria, damaged rough endoplasmic reticula, and damaged columnar epithelial cells in the common bile duct with microvillus shedding (Figure 5J‐L). Thus, the well‐known pathological characteristics of IRI were largely reduced in IFLT grafts.

### 3.5 IFLT prevents transcriptional reprogramming and the activation of sterile inflammation

**FIGURE 6:**
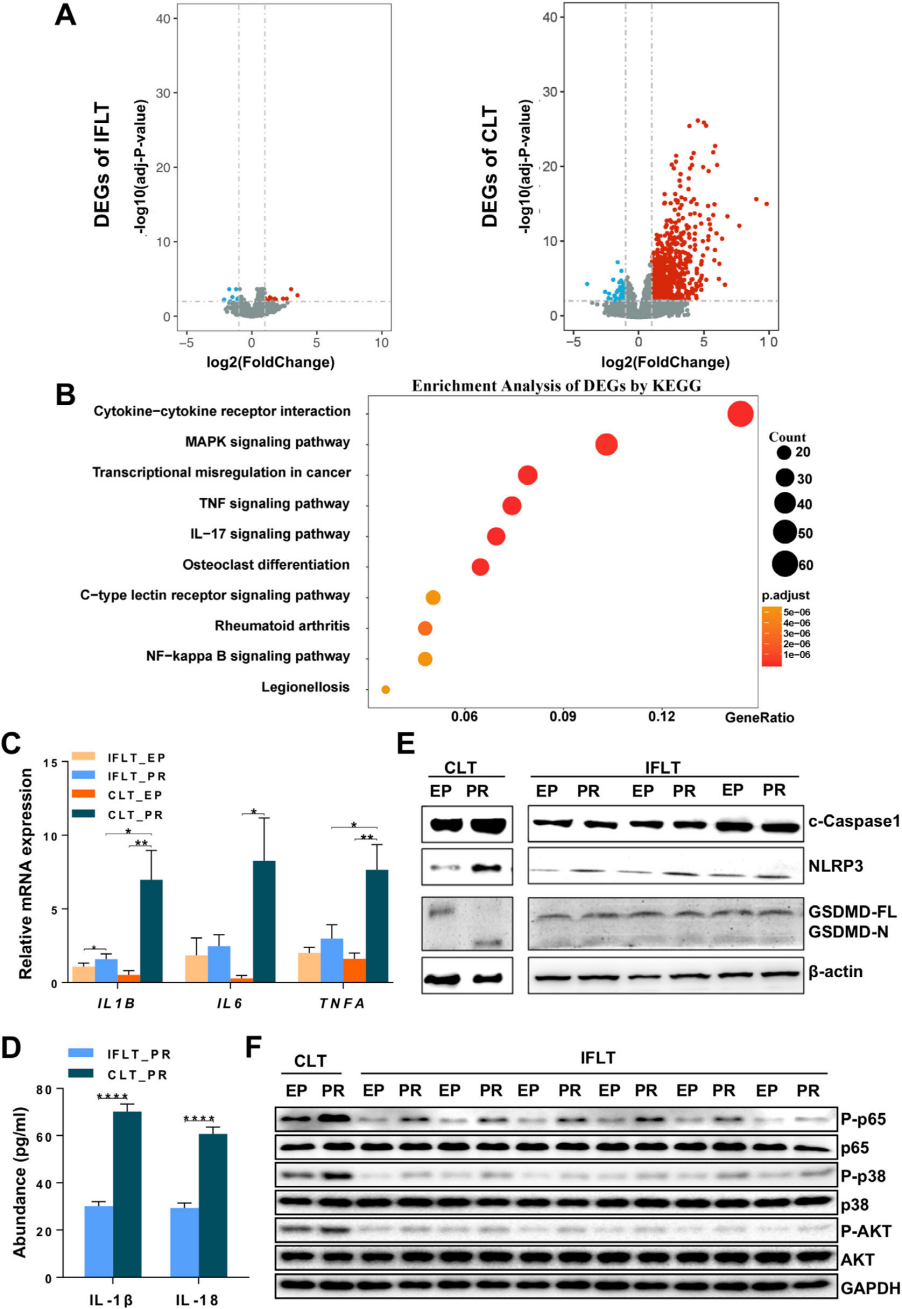
IFLT inhibited gene transcriptional reprogramming and the activation of multiple proinflammatory pathways. (A) The volcano diagram showing that the transcription of only 11 genes was significantly upregulated and downregulated PR in the IFLT group. In contrast, a total of 805 and 58 genes were upregulated and downregulated PR in the CLT group. (B) KEGG pathway analysis of the DEGs revealed the top 10 pathways in the CLT group. (C) The transcription of proinflammatory cytokines (TNF‐α, IL‐1β and IL‐6) in liver graft biopsies was analyzed by RT‐qPCR. (D) The release of IL‐1β and IL‐18 in liver biopsies PR in the two groups was analyzed by ELISA. (E) Western blot analysis of the activation of NLRP3 and Caspase‐1, as well as the cleavage of GSDMD, in liver biopsies. (F) Western blot analysis of Akt, NF‐κB and MAPK in the two groups. Abbreviations: CLT, conventional liver transplantation; EP, end‐preservation; IFLT, ischemia‐free liver transplantation; PR, postrevascularization

Sharp metabolic changes can induce profound transcriptional reprogramming during liver transplantation.^3^ Transcriptome analysis of the liver biopsies showed 805 significantly upregulated genes and 58 downregulated genes in the CLT grafts. Strikingly, there were only 11 upregulated genes and 11 downregulated genes in the IFLT grafts (Figure 6A). The expression of the top five DEGs with the smallest *p* values in the IFLT and CLT groups was verified by RT‐qPCR (Figure S1 and Table S5). These data suggest that the IFLT procedure does not trigger transcriptional reprogramming.

IRI often causes profound sterile inflammation in reperfused grafts.^18^ KEGG pathway analysis revealed that among the top 10 pathways with significant DEG enrichment, five inflammatory pathways, including “cytokine‐cytokine receptor interaction,” “mitogen‐activated protein kinase (MAPK) signaling pathway,” “tumor necrosis factor (TNF) signaling pathway,” “IL‐17 signaling pathway,” and “nuclear factor kappa B (NF‐κB) signaling pathway,” were identified in the CLT group (Figure 6B). There were 59 DEGs enriched in the “cytokine‐cytokine receptor interaction” pathway, which was also the top one distinct pathway in the liver biopsies obtained PR between the CLT and IFLT grafts (Figure S2). Of the proinflammatory cytokines associated with graft IRI, TNF‐α, IL‐1β, and IL‐6 are among the most important.^1^ The transcription of these cytokines was not increased in the IFLT group, while significant increases in the transcription of these cytokines were observed in the CLT group (Figure 6C). The maturation of IL‐1β is tightly controlled by the inflammasome complex, which leads to activation of the IL‐1β‐converting enzyme caspase‐1. Activated Caspase‐1 then cleaves gasdermin D (GSDMD) to form membrane pores to release proinflammatory IL‐1β and IL‐18.^19^ We found significant release of IL‐1β and IL‐18 (Figure 6D), activation of the inflammasome sensors NOD‐like receptor protein‐3 (NLRP3) and Caspase‐1, and cleavage of GSDMD (Figure 6E) in liver biopsies obtained PR in the CLT group. In contrast, activation of the inflammasome complex and pyroptosis were almost absent in the IFLT group. In addition, studies have shown that the MAPK, Akt and NF‐κB pathways are key players in IRI.^20^, ^21^ We validated that these proinflammatory pathways were hardly activated by phosphorylation in the IFLT grafts as they were in the CLT grafts (Figure 6F).

### 3.6 Proteomics analysis reveals distinct metabolic and immunological characteristics between IFLT and CLT

**FIGURE 7:**
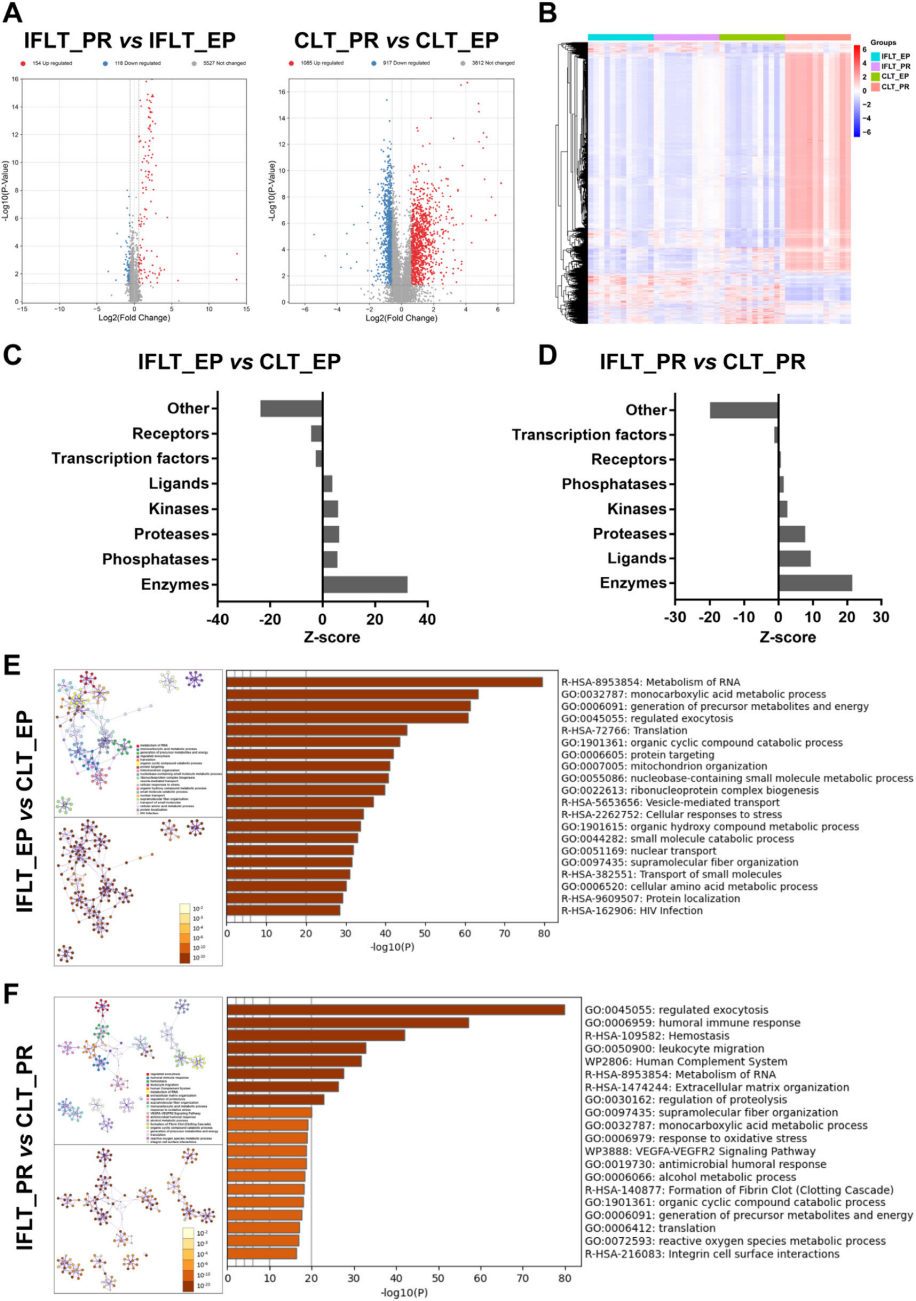
IFLT maintains stable expression of metabolic enzymes and neutrophil function. (A) Volcano diagram showing the deferentially expressed proteins (DEPs) in the IFLT_EP and IFLT_PR groups and in the CLT_EP and CLT_PR groups. (B) Hierarchical clustering heat map showing all detected proteins in the four groups. (C and D) The “Enrichment by Protein Function” tool of MetaCore was used to enrich DEPs by objects from different protein classes in the IFLT_EP and CLT_EP groups (C) and in the IFLT_PR and CLT_PR groups (D). (E and F) The functional enrichment analysis of DEPs using Metascape revealed the top 20 functions in the IFLT_EP and CLT_EP groups (E) and in the IFLT_PR and CLT_PR groups (F) (upper left: color by cluster, as listed on the right; lower left: color by *p* value). |fold change| > 1.5, *p *< 0.05. Abbreviations: CLT, conventional liver transplantation; DEPs, differentially expressed proteins; EP, end‐preservation; IFLT, ischemia‐free liver transplantation; PR, postrevascularization

To further confirm the transcriptome profiling results, proteomics analysis was conducted. A total of 1085 proteins were significantly upregulated, and 917 proteins were downregulated in the CLT group. In contrast, there were only 154 upregulated proteins and 118 downregulated proteins in the IFLT group (Figure 7A). A hierarchical clustering heat map of all identified proteins in the four groups showed significant differences between CLT_PR and CLT_EP samples (Figure 7B). Using quantitative values from proteomics data, PCA and OPLS‐DA also indicated more differences in the CLT versus IFLT group (Figure S3). According to the Z‐scores of different protein classes, enzymes were the most important differentially expressed proteins (DEPs) between the IFLT and CLT groups (Figure 7C, D). Functional enrichment analysis of the DEPs using Metascape showed that “metabolism of RNA” was the major differentially expressed process between IFLT_EP and CLT_EP samples (Figure 7E), and “regulated exocytosis” and “humoral immune response” were among the major differentially expressed processes between IFLT_PR and CLT_PR samples (Figure 7F). Combined analysis of DEGs and DEPs using MetaCore showed that four of the top 10 Gene Ontology (GO) processes between IFLT_EP and CLT_EP samples were metabolic processes, while “regulated exocytosis” and “neutrophil‐mediated immunity” were also among the major differentially expressed processes between IFLT_PR and CLT_PR samples (Figure S4A), which was consistent with the functional enrichment analysis of DEPs.

### 3.7 Combined analysis of transcriptomics, proteomics, and metabolomics using MetaCore

Combined analysis of DEGs, DEPs, and DEMs in the IFLT_EP and CLT_EP groups by MetaCore showed that the top 10 metabolic networks mainly included amino acid (alanine, glycine, cysteine, glutamic acid, serine, methionine, ornithine, etc.) and lipid (glycosphingolipid and triacylglycerol) metabolism (Figure S4B,C). Among these pathways, the top one was glycine and L‐serine metabolism.

**FIGURE 8:**
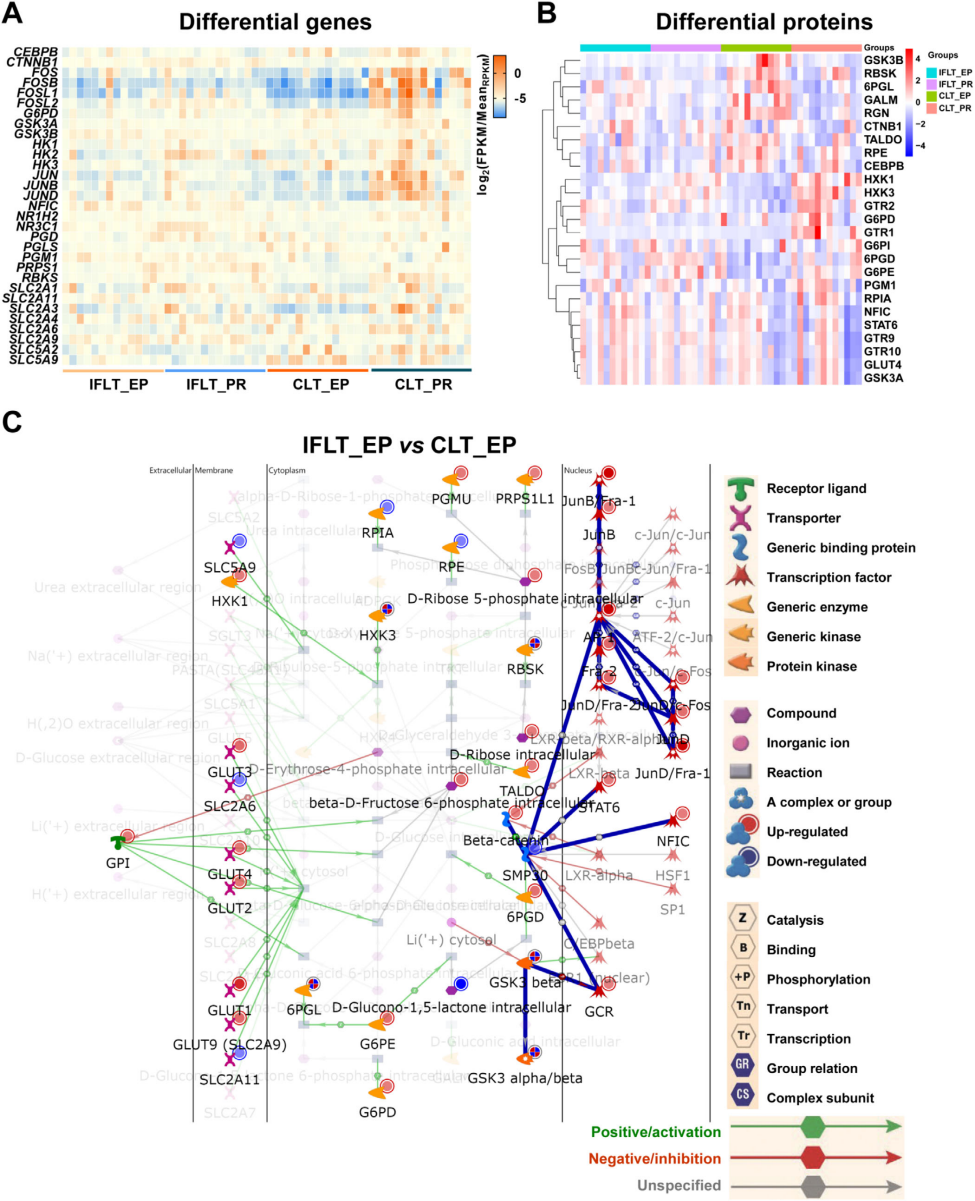
Transcriptome, proteome, and metabolome enrichment of the pentose phosphate pathway and transport. (A and B) Heat map showing 31 differentially expressed genes (DEGs) (A) and 25 differentially expressed proteins (DEPs) (B) in the “pentose phosphate pathways and transport” metabolic network (endogenous). (C) Metabolic network (endogenous) of pentose phosphate and transport. Differential genes, proteins, and metabolites in the network were analyzed using MetaCore. Differential genes, proteins, and metabolites, *p* < 0.05. Abbreviations: CLT, conventional liver transplantation; EP, end‐preservation; IFLT, ischemia‐free liver transplantation

Particularly, in line with the metabolomics results, gene transcription and protein expression in the “PPPs and transport” metabolic network were stable throughout IFLT. In contrast, a number of these genes were downregulated during preservation and upregulated PR in the CLT group (Figure 8A). A hierarchical clustering heat map of 25 DEPs between the four groups in the network is shown in Figure 8B.

The “PPPs and transport” metabolic network identified by the combined analysis of transcriptome, proteomics, and metabolomics data using MetaCore is shown in Figure 8C. There were eight genes and their encoded proteins differentially expressed in the IFLT_EP versus CLT_EP group. Expression of the genes glucose transporter type 9 (*GLUT9*), nuclear factor I C (*NFIC*), phosphoglucomutase 1 (*PGM1*), and catenin beta 1 (*CTNNB1*) and their encoded proteins were upregulated. The expression of hexokinase 3 (*HXK3*) and 6‐phosphogluconolactonase (*6PGL*) was downregulated at gene transcription level but upregulated at protein expression level. The expression of glycogen synthase kinase 3 beta (*GSK3B*) and ribokinase (*RBSK*) was upregulated at gene transcription level but downregulated at protein expression level.

Glucose transporter type 3 (GLUT3) and GLUT9 transport extracellular D‐glucose into cells. PGMU catalyzes the biotransformation of alpha‐D‐ribose‐1‐phosphate to D‐ribose 5‐phosphate. RBKS catalyzes the biotransformation of adenosine triphosphate (ATP) and D‐ribose to D‐ribose 5‐phosphate and adenosine diphosphate (ADP). These key players of the PPP, including GLUT9, PGMU, D‐ribose, and D‐ribose 5‐phosphate, were all upregulated in the IFLT_EP versus CLT_EP group.

### 3.8 Local immunity and systemic inflammation were suppressed in IFLT grafts

**FIGURE 9:**
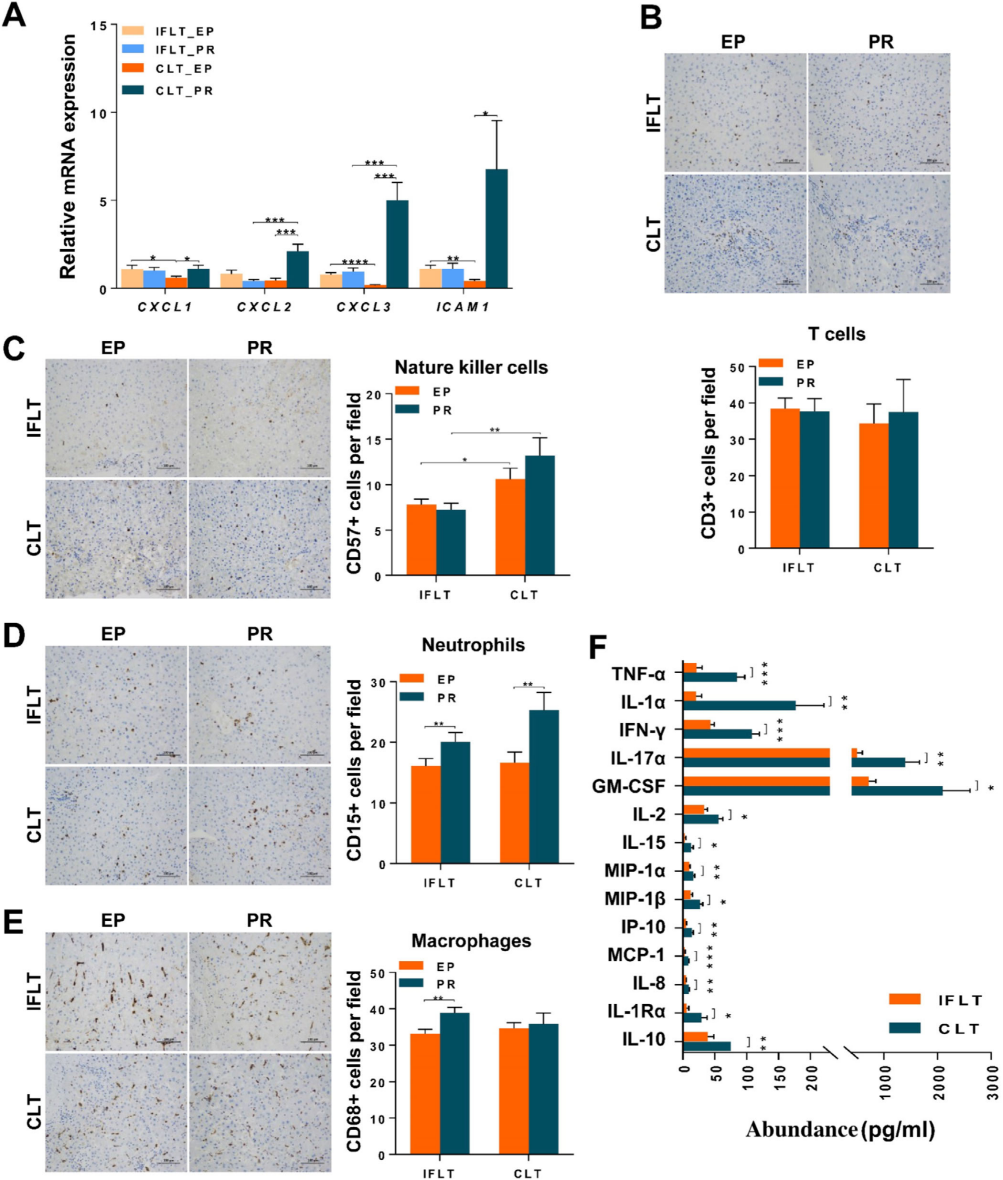
IFLT suppressed local and systemic immunity. (A) The production of chemokines (CXCL‐1, CXCL‐2, CXCL‐3 and ICAM‐1) in liver graft biopsies was analyzed by RT‐qPCR. (B‐E) The number of nucleated T cells (CD3+), NK cells (CD57+), neutrophils (CD15+) or macrophages (CD68+) in each area was counted and is expressed as the number of cells per field. In total, 38 patients who underwent CLT and 38 patients who underwent IFLT were included. Samples were collected at the end of preservation (EP) and one hour after graft revascularization (PR).(F) The different levels of 14 cytokines and cytokine receptors in the recipient serum at postoperative day 1 in the two groups are shown. Two‐tailed Student’s t‐test was used for statistical analysis; *P < 0.05, **P < 0.01, ***P < 0.001. IFLT, ischemia‐free liver transplantation; CLT, conventional liver transplantation; EP, end‐preservation; PR, postrevascularization.

**FIGURE 10:**
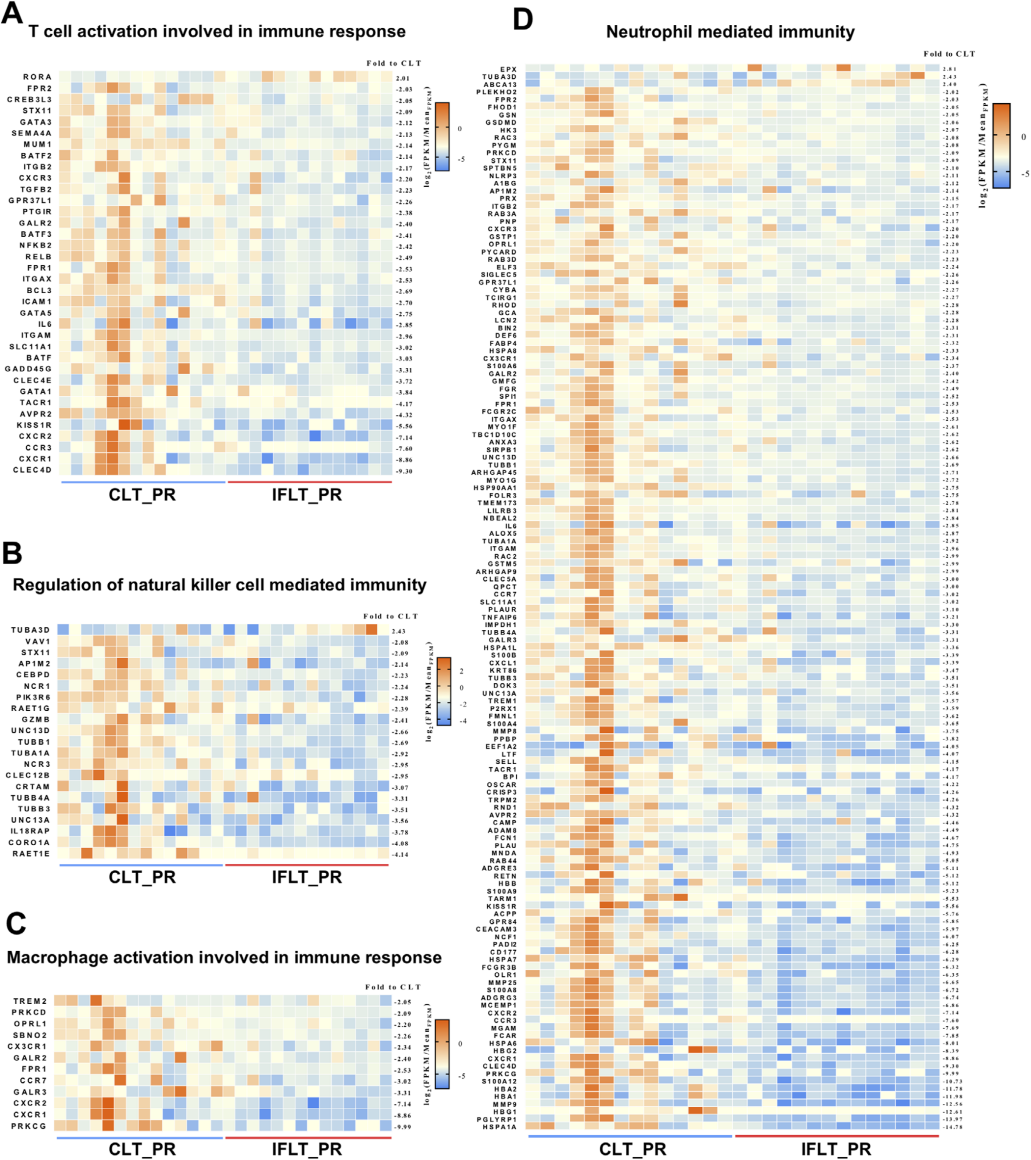
Heat maps showing four immune‐related Gene Ontology (GO) processes.Differentially expressed genes (DEG) enrichment in four GO processes, including “T cell activation involved in immune response” (A), “regulation of natural killer cell‐mediated immunity” (B), “macrophage activation involved in immune response” (C) and “neutrophil‐mediated immunity” (D), using the MetaCore database. IFLT, ischemia‐free liver transplantation; CLT, conventional liver transplantation; PR, postrevascularization.

Sterile inflammation subsequently recruits and activates innate and acquired immune cells during IRI.^3^ The transcription levels of the chemokines C‐X‐C motif ligand‐1 (CXCL1), CXCL2, and CXCL3 were upregulated in the CLT group (Figure 9A). In contrast, the transcription levels of these chemokines were stable in the IFLT group. The number of T cells was comparable between the two groups (Figure 9B). The number of natural killer (NK) cells in the grafts PR was lower in the IFLT versus CLT group (Figure 9C). A more obvious increase in neutrophils PR was documented in the CLT group when compared to the IFLT group (Figure 9D). Unexpectedly, the number of macrophages increased PR in the IFLT grafts instead of the CLT grafts (Figure 9, 10).

Transcriptome analysis showed that expression of a number of genes involved in T cell activation, macrophage activation, NK cell‐mediated immunity, and neutrophil‐mediated immunity was enhanced after PR in the CLT group in comparison to the IFLT group (). Furthermore, significantly higher levels of 14 cytokines, including IL‐1, IL‐1RA, IL‐2, IL‐8, IL‐10, IL‐15, IL‐17A, TNF‐α, interferon (IFN)‐γ, interferon‐inducible protein (IP)‐10, monocyte chemoattractant protein (MCP)‐1, and granulocyte‐macrophage colony stimulating factor (GM‐CSF), were found in the serum of recipients on day 1 posttransplantation in the CLT versus IFLT group (Figure 9F). Collectively, these results suggest a largely suppressed local and systemic inflammation in IFLT.

## 4 DISCUSSION

During conventional transplant procedures in all types of solid organ transplantation, oxygenated blood supply is completely interrupted throughout organ procurement, *ex situ* preservation, and implantation. After revascularization, the graft can definitely suffer subsequent IRI. However, the pathophysiological complexity of this event remains only poorly understood and is complicated by the concurrence of the allograft rejection response.^1^ Our IFLT approach is an entirely novel approach with no graft ischemia, providing unique clinical and research opportunities for investigating the mechanisms of IRI. In the current study, we examined the pathological, transcriptome, proteome, metabolome, and immunological profiles of IFLT and CLT grafts to provide a landscape on how IFLT might impact graft IRI.

A key pathological characteristic of graft IRI is the presence of many kinds of cell death, including necrosis, apoptosis, and pyroptosis.^22^, ^23^, ^24^ The Suzuki score based on HE staining and apoptosis analysis of liver graft biopsies suggested an almost complete absence of necrosis and apoptosis in IFLT grafts. In addition, inflammasome‐mediated pyroptosis was also largely reduced. Moreover, the TEM results also revealed optimal preservation of the ultrastructure of the grafts in the IFLT group. Collectively, these comprehensive pathological data show for the first time that, by using IFLT, graft IRI can be largely avoided. However, the AST/ALT levels were still increased post‐IFLT. Our previous study showed that liver enzyme levels were slightly increased during *ex situ* NMP,^25^ suggesting that NMP technology itself might also induce graft injury, although a continuous blood supply can be maintained during the procedure. Recently, we documented normal AST/ALT levels on day 1 post‐IFLT in one patient. Therefore, further modifying this procedure might eventually result in a complete avoidance of graft IRI.

During CLT, grafts were subjected to hypothermic and anaerobic metabolism due to the interruption of the blood supply. In sharp contrast, the grafts maintained normothermic and aerobic metabolism throughout IFLT. The metabolomics analysis showed that the metabolic activities of amino acids, lipids, and carbohydrates were suppressed in CLT grafts compared with IFLT grafts. The suppressed functions of a large number of metabolic enzymes identified by the proteomics analysis (Tables S6, S7) might explain the distinct metabolomics profiles. Importantly, we found that the most pronounced metabolic pathway distinguishing CLT from IFLT grafts in both the EP and PR stages was the PPP. Reactive oxygen species (ROS) are the key players in liver IRI, leading to cell death and ultrastructural damage.^1^ ROS detoxification requires the maintenance of GSH by NADPH generated by the PPP. Studies have shown that its rate‐limiting enzyme, glucose‐6‐phosphate dehydrogenase (G6PDH), can protect the brain and heart from IRI.^26^, ^27^ Furthermore, the PPP is the key to maintaining mitochondrial function.^28^ Impaired mitochondrial function plays an essential role in allograft IRI.^29^ In this study, transcriptome, proteome, and metabolome network analysis confirmed that PPP activity was stable throughout IFLT, while it was suppressed during preservation but enhanced postrevascularization in CLT. Consistently, ROS production was enhanced, and the antioxidants GSH and SOD were diminished PR in CLT grafts, while redox hemostasis was maintained in IFLT grafts. These results indicate that IFLT might prevent oxidative stress in grafts by maintaining stable antioxidative PPP activity. However, it is still unknown how IFLT might affect mitochondrial function.

To adapt to anaerobic metabolism, significant transcriptional reprogramming occurs during liver transplantation.^3^ In the current study, transcriptome analysis confirmed this phenomenon in the CLT group, while it was largely prevented in the IFLT group. Importantly, transcriptome analysis showed that several inflammatory pathways, including the NF‐κB, MAPK, TNF‐α, and IL‐17 pathways, with large numbers of DEGs were activated in the CLT group and were almost inactivated in the IFLT group. These results were further confirmed by RT‐qPCR, Western blotting and ELISA. It has been reported that these pathways are key mediators and potential therapeutic targets of graft IRI.^30^, ^31^, ^32^ However, it is almost impossible to simultaneously target all of these pathways by either pharmacological methods or gene therapy. In addition, intragraft sterile inflammation is a well‐known characteristic that shapes the long‐term immune microenvironment during IRI.^33^ Studies have shown that sterile inflammation is associated with chronic graft loss.^34^ Therefore, IFLT might provide benefits for long‐term graft survival.

Importantly, sterile inflammation during IRI may not only damage the graft itself but also initiate the recruitment of innate and adaptive immune cells, leading to augmented alloimmunity and more frequent rejection.^35^ It is well known that neutrophils, macrophages and NK cells all play critical roles in the pathogenesis of graft IRI.^36^, ^37^, ^38^ We showed decreased levels of chemokines by RT‐qPCR and reduced numbers of NK cells and neutrophils by immunohistochemical staining, as well as reduced activity of NK cells, macrophages and neutrophils by transcriptome analysis, in IFLT grafts compared to CLT grafts. Proteome analysis also showed that neutrophil‐mediated immunity was enhanced in CLT grafts compared to IFLT grafts. However, we did not find a significantly reduced incidence of allograft rejection in the IFLT group,^16^ probably due to the use of powerful immunosuppressive drugs and the limited sample size. Rebecca et al reported that exposure of the liver to IRI may subsequently cause inflammatory responses in other organs, which might lead to multiorgan failure.^39^ Indeed, we documented significant decreases in 14 cytokines in the recipients’ serum on day 1 posttransplantation in the IFLT versus CLT group. Considering the profound differences in intragraft immunity and systemic inflammation early posttransplantation, it will be of great importance to investigate how IFLT alters the incidence and characteristics of allograft rejection in an animal model.

There are some weaknesses in the current study. First, due to the limited samples collected, we could not conduct all the analyses in all the patients included in the clinical trial, which might lead to sample selection bias. Second, this study aimed to outline the comprehensive impacts of IFLT on the pathogenesis of IRI. Future studies should focus on specific mechanisms. Finally, all the findings were from human samples, and the gain or loss of function experiments in animal studies is pending.

In conclusion, these results show that IFLT is an efficient approach to minimize IRI, which might improve transplant outcomes and increase the availability of organs for transplantation. In addition, this novel approach provides a unique model for investigating the molecular mechanism of graft IRI and elucidating the interaction between IRI and allograft rejection.

## ETHICS APPROVAL AND CONSENT TO PARTICIPATE

The study protocol (ChiCTR‐OPN‐17012090) was carried out in compliance with institutional and governmental requirements approved by the Ethics Committee of the First Affiliated Hospital, Sun Yat‐sen University, Guangzhou, China.

## CONFLICT OF INTEREST

The authors declare that they have no competing interests.

## AUTHOR CONTRIBUTIONS

Xiaoshun He,Zhiyong Guo, Jinghong Xu, Shanzhou Huang, and Meixian Yin contributed to the conceptualization and trial design. Zhiyong Guo, Jinghong Xu, Shanzhou Huang, Meixian Yin, Kunpeng Liu, Yuting Lu, and Haitian Chen managed the samples and performed relevant experiments. Qiang Zhao, Weiqiang Ju, Dongping Wang, Ningxin Gao, Changjun Huang, Lu Yang, Maogen Chen, Linhe Wang, Caihui Zhu, Yixi Zhang, Yunhua Tang, and Zhiheng Zhang. contributed to data collection. Xiaoshun He, Zhiyong Guo, Jinghong Xu, Shanzhou Huang, Meixian Yin, Yi Ma, Anbin Hu, Yinghua Chen, and Xiaofeng Zhu analyzed and interpreted the data. Zhiyong Guo, Jinghong Xu, Shanzhou Huang, and Meixian Yin drafted the manuscript, which was critically reviewed and revised by Xiaoshun He, Zhiyong Guo, Anbin Hu, Yinghua Chen and Xiaofeng Zhu. Zhiyong Guo, Jinghong Xu, Shanzhou Huang, and Meixian Yin contributed equally to the work and should be considered first coauthors. All authors reviewed and approved the final manuscript.

## Supporting information

SUPPORTING INFORMATION

## Data Availability

All data associated with this study are presented in the paper or the Supplementary Materials. The GEO accession number for the microarray data reported in this paper is GEO: GSE113024.

## References

[1] de Rougemont O , Dutkowski P , Clavien PA . Biological modulation of liver ischemia‐reperfusion injury. Curr Opin Organ Transplant. 2010;15(2):183–189. 20125019 10.1097/MOT.0b013e3283373ced

[2] Nemes B , Gámán G , Polak WG , et al. Extended‐criteria donors in liver transplantation Part II: reviewing the impact of extended‐criteria donors on the complications and outcomes of liver transplantation. Expert Rev Gastroent. 2016;10(7):841–859. 10.1586/17474124.2016.1149062 26831547

[3] Eltzschig HK , Eckle T . Ischemia and reperfusion–from mechanism to translation. Nat Med. 2011;17(11):1391–1401. 22064429 10.1038/nm.2507 PMC3886192

[4] Bruinsma BG , Yeh H , Ozer S , et al. Subnormothermic machine perfusion for ex vivo preservation and recovery of the human liver for transplantation. Am J Transplant. 2014;14(6):1400–1409. 24758155 10.1111/ajt.12727 PMC4470578

[5] Westerkamp AC , Karimian N , Matton AP , et al. Oxygenated hypothermic machine perfusion after static cold storage improves hepatobiliary function of extended criteria donor livers. Transplantation. 2016;100(4):825–835. 26863473 10.1097/TP.0000000000001081

[6] Burlage LC , Karimian N , Westerkamp AC , et al. Oxygenated hypothermic machine perfusion after static cold storage improves endothelial function of extended criteria donor livers. HPB (Oxford). 2017;19(6):538–546. 28351756 10.1016/j.hpb.2017.02.439

[7] Ceresa CDL , Nasralla D , Knight S , Friend PJ . Cold storage or normothermic perfusion for liver transplantation: probable application and indications. Curr Opin Organ Transplant. 2017;22(3):300–305. 28301388 10.1097/MOT.0000000000000410

[8] Selten J , Schlegel A , de Jonge J , Dutkowski P . Hypo‐ and normothermic perfusion of the liver: which way to go?. Best Pract Res Clin Gastroenterol. 2017;31(2):171–179. 28624105 10.1016/j.bpg.2017.04.001

[9] von Horn C , Baba HA , Hannaert P , et al. Controlled oxygenated rewarming up to normothermia for pretransplant reconditioning of liver grafts. Clin Transplant. 2017;31(11):e13101. 10.1111/ctr.13101 28871615

[10] Nasralla D , Coussios CC , Mergental H , et al. A randomized trial of normothermic preservation in liver transplantation. Nature. 2018;557(7703):50–56. 29670285 10.1038/s41586-018-0047-9

[11] de Meijer VE , Fujiyoshi M , Porte RJ . Ex situ machine perfusion strategies in liver transplantation. J Hepatol. 2019;70(1):203–205. 30409464 10.1016/j.jhep.2018.09.019

[12] He X , Guo Z , Zhao Q , et al. The first case of ischemia‐free organ transplantation in humans: a proof of concept. Am J Transplant. 2018;18(3):737–744. 29127685 10.1111/ajt.14583

[13] Suzuki S , Toledo‐Pereyra LH , Rodriguez FJ , Cejalvo D . Neutrophil infiltration as an important factor in liver ischemia and reperfusion injury. Modulating effects of FK506 and cyclosporine. Transplantation. 1993;55(6):1265–1272. 7685932 10.1097/00007890-199306000-00011

[14] Wei H , Yin M , Lu Y , et al. Mild hypothermia improves neurological outcome in mice after cardiopulmonary resuscitation through silent information regulator 1‐actviated autophagy. Cell Death Discov. 2019;5:129. 31428461 10.1038/s41420-019-0209-z PMC6690976

[15] Huang S , Ju W , Zhu Z , et al. Comprehensive and combined omics analysis reveals factors of ischemia‐reperfusion injury in liver transplantation. Epigenomics‐UK. 2019;11(5):527–542. 10.2217/epi-2018-0189 30700158

[16] Guo Z , Zhao Q , Huang S , Huang C , Zhang J . Liver transplantation without graft ischemia in humans. Lancet Reg Health West Pac. 2021. 10.1101/2020.04.20.20065979 PMC8406025 34590063

[17] Quaegebeur A , Segura I , Schmieder R , et al. Deletion or inhibition of the oxygen sensor PHD1 protects against ischemic stroke via reprogramming of neuronal metabolism. Cell Metab. 2016;23(2):280–291. 26774962 10.1016/j.cmet.2015.12.007 PMC4880550

[18] Chen GY , Nuñez G . Sterile inflammation: sensing and reacting to damage. Nat Rev Immunol. 2010;10(12):826–837. 21088683 10.1038/nri2873 PMC3114424

[19] Newman AM , Liu CL , Green MR , et al. Robust enumeration of cell subsets from tissue expression profiles. Nat Methods. 2015;12(5):453–457. 25822800 10.1038/nmeth.3337 PMC4739640

[20] Kamo N , Ke B , Busuttil RW , Kupiec‐Weglinski JW . PTEN‐mediated Akt/β‐catenin/Foxo1 signaling regulates innate immune responses in mouse liver ischemia/reperfusion injury. Hepatology. 2013;57(1):289–298. 22807038 10.1002/hep.25958 PMC3524373

[21] Duarte S , Shen XD , Fondevila C , Busuttil RW , Coito AJ . Fibronectin‐α4β1 interactions in hepatic cold ischemia and reperfusion injury: regulation of MMP‐9 and MT1‐MMP via the p38 MAPK pathway. Am J Transplant. 2012;12(10):2689–2699. 22812390 10.1111/j.1600-6143.2012.04161.x PMC3459169

[22] Pefanis A , Ierino FL , Murphy JM , Cowan PJ . Regulated necrosis in kidney ischemia‐reperfusion injury. Kidney Int. 2019;96(2):291–301. 31005270 10.1016/j.kint.2019.02.009

[23] Ye L , He S , Mao X , Zhang Y , Cai Y , Li S . Effect of hepatic macrophage polarization and apoptosis on liver ischemia and reperfusion injury during liver transplantation. Front Immunol. 2020;11:1193. 32676077 10.3389/fimmu.2020.01193 PMC7333353

[24] Weigt SS , Palchevskiy V , Belperio JA . Inflammasomes and IL‐1 biology in the pathogenesis of allograft dysfunction. J Clin Invest. 2017;127(6):2022–2029. 28569730 10.1172/JCI93537 PMC5451233

[25] Zhang Z , Tang Y , Zhao Q , et al. Association of perfusion characteristics and posttransplant liver function in ischemia‐free liver transplantation. Liver Transplantation. 2020;26(11):1441–1454. 32542994 10.1002/lt.25825

[26] Cao L , Zhang D , Chen J , et al. G6PD plays a neuroprotective role in brain ischemia through promoting pentose phosphate pathway. Free Radical Bio Med. 2017;112:433–444. 28823591 10.1016/j.freeradbiomed.2017.08.011

[27] Jain M , Cui L , Brenner DA , et al. Increased myocardial dysfunction after ischemia‐reperfusion in mice lacking glucose‐6‐phosphate dehydrogenase. Circulation. 2004;109(7):898–903. 14757696 10.1161/01.CIR.0000112605.43318.CA

[28] Zeng S , Zhao Z , Zheng S , et al. The E3 ubiquitin ligase TRIM31 is involved in cerebral ischemic injury by promoting degradation of TIGAR. Redox Biol. 2021;45:102058. 34218200 10.1016/j.redox.2021.102058 PMC8260875

[29] Karangwa S , Panayotova G , Dutkowski P , Porte RJ , Guarrera JV , Schlegel A . Hypothermic machine perfusion in liver transplantation. Int J Surg. 2020;82s:44–51. 32353556 10.1016/j.ijsu.2020.04.057

[30] Steenbergen C . The role of p38 mitogen‐activated protein kinase in myocardial ischemia/reperfusion injury; relationship to ischemic preconditioning. Basic Res Cardiol. 2002;97(4):276–285. 12111037 10.1007/s00395-002-0364-9

[31] Tan Z , Jiang R , Wang X , et al. RORγt+IL‐17+ neutrophils play a critical role in hepatic ischemia‐reperfusion injury. J Mol Cell Biol. 2013;5(2):143–146. 23362310 10.1093/jmcb/mjs065 PMC3934999

[32] Schulz R , Aker S , Belosjorow S , Heusch G . TNFalpha in ischemia/reperfusion injury and heart failure. Basic Res Cardiol. 2004;99(1):8–11. 14685700 10.1007/s00395-003-0431-x

[33] van Golen RF , Reiniers MJ , Olthof PB , van Gulik TM , Heger M . Sterile inflammation in hepatic ischemia/reperfusion injury: present concepts and potential therapeutics. J Gastroenterol Hepatol. 2013;28(3):394–400. 23216461 10.1111/jgh.12072

[34] Khan F , Sar A , Gonul I , et al. Graft inflammation and histologic indicators of kidney chronic allograft failure: low‐expressing interleukin‐10 genotypes cannot be ignored. Transplantation. 2010;90(6):630–638. 20622753 10.1097/TP.0b013e3181ea391e

[35] Otterbein LE , Fan Z , Koulmanda M , Thronley T , Strom TB . Innate immunity for better or worse govern the allograft response. Curr Opin Organ Transplant. 2015;20(1):8–12. 25563986 10.1097/MOT.0000000000000152 PMC4374347

[36] Oliveira THC , Marques PE , Proost P , Teixeira MMM . Neutrophils: a cornerstone of liver ischemia and reperfusion injury. Lab Invest. 2018;98(1):51–62. 28920945 10.1038/labinvest.2017.90

[37] Li L , Okusa MD . Macrophages, dendritic cells, and kidney ischemia‐reperfusion injury. Semin Nephrol. 2010;30(3):268–277. 20620671 10.1016/j.semnephrol.2010.03.005 PMC2904394

[38] Ware R , Kumar V . Complexity and function of natural killer T cells with potential application to hepatic transplant survival. Liver Transplant. 2017;23(12):1589–1592. 10.1002/lt.24950 PMC6075735 28945950

[39] Sosa RA , Zarrinpar A , Rossetti M , et al. Early cytokine signatures of ischemia/reperfusion injury in human orthotopic liver transplantation. JCI Insight. 2016;1(20):e89679. 27942590 10.1172/jci.insight.89679 PMC5135282

