# Supplementary material for: Abrogation of graft ischemia‐reperfusion injury in ischemia‐free liver transplantation
Source: Clin Transl Med. 2022 Apr 26;12(4):e546. doi: 10.1002/ctm2.546 (PMC9042797; doi:10.1002/ctm2.546)
Supplement: Supplementary file 1 — SUPPORTING INFORMATION [file CTM2-12-e546-s001.docx]

**SUPPORTING INFORMATION**

**Fig. S1. The verification of RT-qPCR of top five differential expressed genes with the smallest *P* values in IFLT_PR vs IFLT_EP (left) and CLT_PR vs CLT_EP (right).** IFLT, ischemia-free liver transplantation; CLT, conventional liver transplantation; EP, end-preservation; PR, postrevascularization. **P* < 0.05, ***P* < 0.01, ****P* < 0.001, *****P* < 0.0001.

**Fig. S2. Heatmap showing the DEGs enrichment in the cytokine-cytokine receptor pathway.** IFLT, ischemia-free liver transplantation; CLT, conventional liver transplantation; DEGs, defferentially expressed genes; EP, end-preservation; PR, postrevascularization.


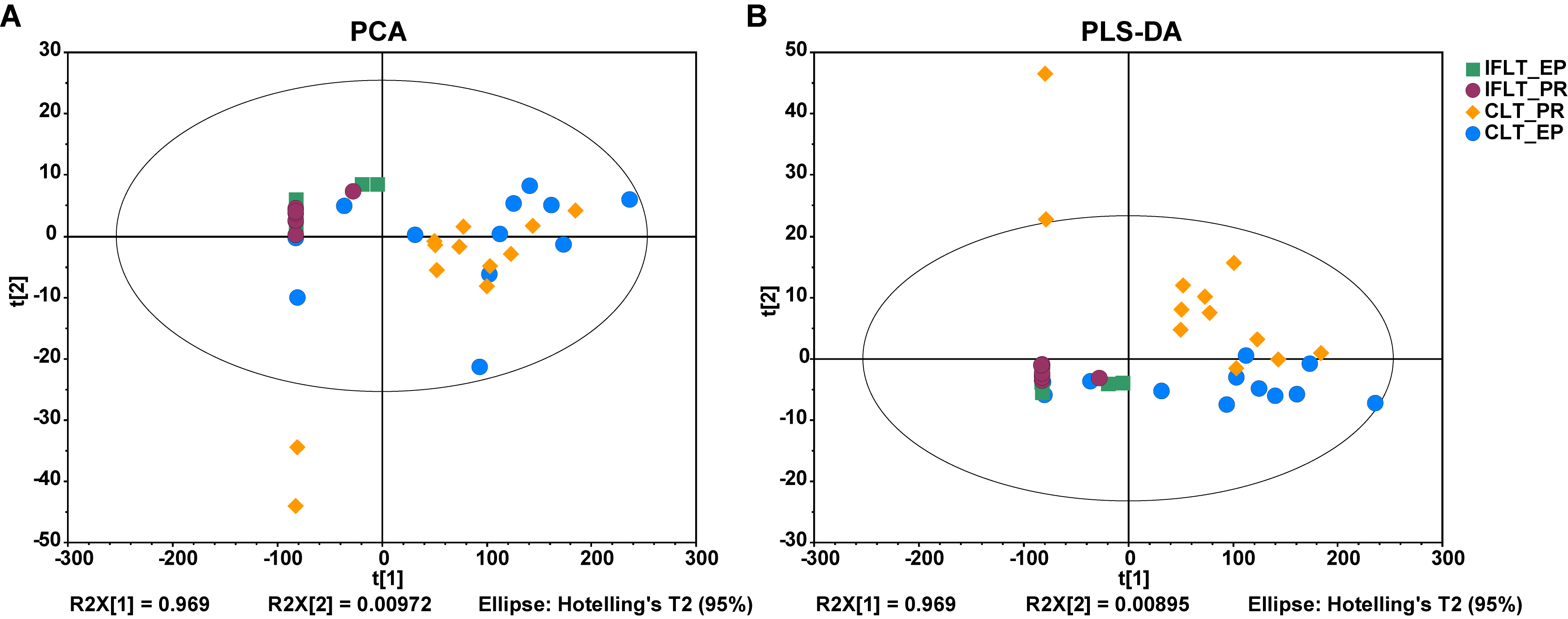


**Fig. S3. PCA and PLS-DA for proteome.** (A) Principal component analysis (PCA) and (B) orthogonal partial least squares-discriminant analysis (OPLS-DA) using quantitative values from proteome data.


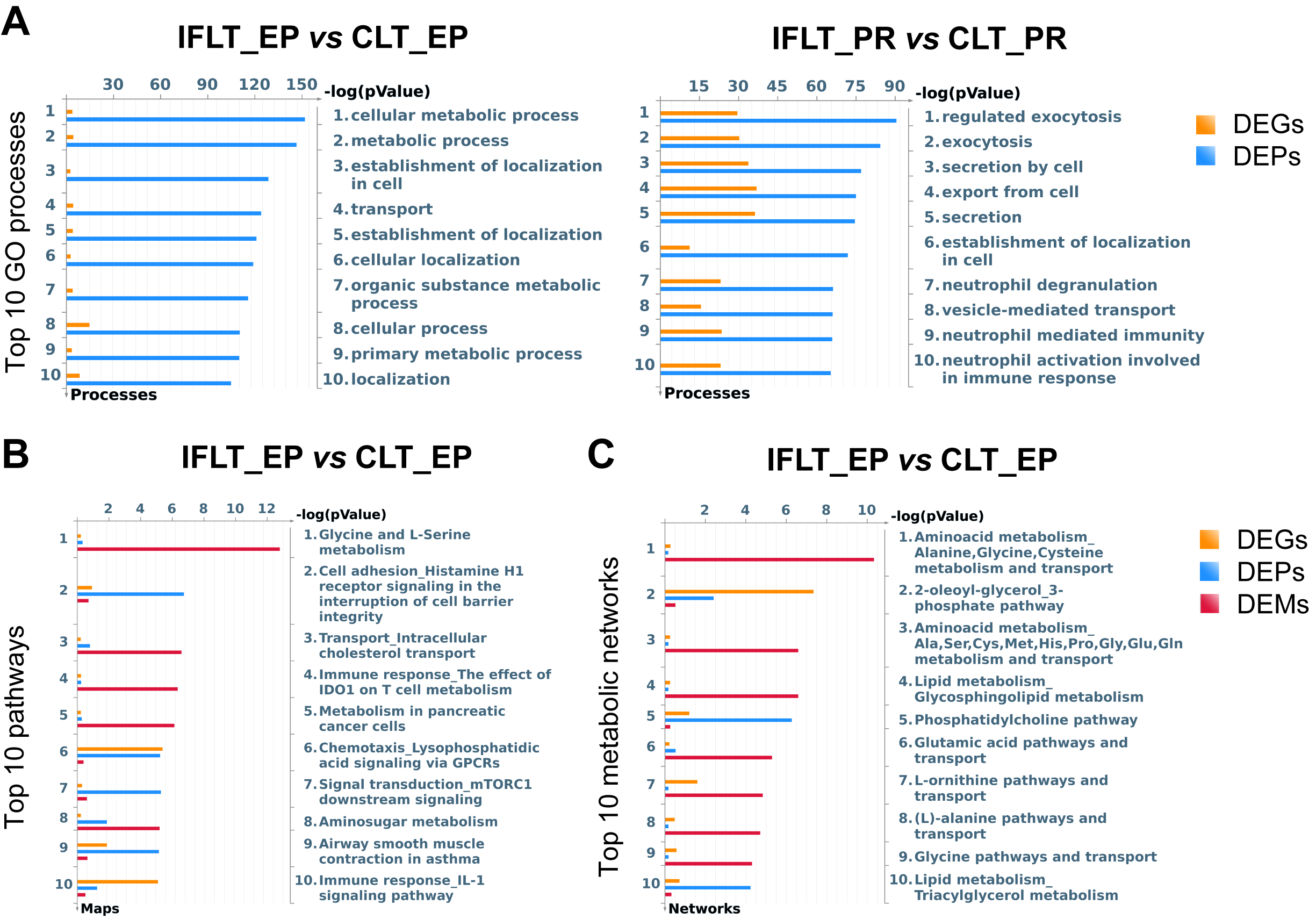


**Fig. S4. Multi-omic analysis**. (A) GO processes analysis of DEGs and DEPs was performed with the MetaCore. (B and C) Pathways analysis (B) and metabolic networks (C) of DEGs, DEPs and DEMs were performed with the MetaCore. DEPs, differentially expressed genes, |fold change| >2, *P* < 0.05. DEPs, differentially expressed proteins, |fold change| >1.5, *P* < 0.05. DEMs, differentially expressed metabolites, *P* < 0.05. IFLT, ischemia-free liver transplantation; CLT, conventional liver transplantation; EP, end-preservation; PR, postrevascularization.

**Table S1. Sequence of primers used for quantitative real-time** [**polymerase chain reaction (RT-qPCR)**](http://www.baidu.com/link?url=NvEPIDZkrX-QTb4jVUbRQErOffbTEt2noYYd7JFLABfW9yD58bHjn9N0ocZPwTLeyXK1qUUZpRnaVsqWTQy9Rq) **in the study.**

| **Genes** | **Sequence (5'-3')** |
| --- | --- |
| CXCL-1-S | CTCTTCCGCTCCTCTCACAG |
| CXCL-1-A | GGGGACTTCACGTTCACACT |
| CXCL-2-S | CTCAAGAATGGGCAGAAAGC |
| CXCL-2-A | AAACACATTAGGCGCAATCC |
| CXCL-3-S | CGCCCAAACCGAAGTCATAG |
| CXCL-3-A | GCTCCCCTTGTTCAGTATCTTTT |
| ICAM-1-S | CAATGTGCTATTCAAACTGCCC |
| ICAM-1-A | CAGCGTAGGGTAAGGTTCTTG |
| IL-1β-S | CAAGCTGAGGAAGATGCTGGTT |
| IL-1β-A | CCACTTGTTGCTCCATATCCTGT |
| IL-6-S | AGTAGTGAGGAACAAGCCAGAGC |
| IL-6-A | TACATTTGCCGAAGAGCCCTC |
| TNF-α-S | AGCCTCTTCTCCTTCCTGATCG |
| TNF-α-A | TTGCTACAACATGGGCTACAGG |
| β-actin-S | AGCGAGCATCCCCCAAAGTT |
| β-actin-A | GGGCACGAAGGCTCATCATT |

CXCL, chemokine (C-X-C motif) ligand; IL-1β, Interleukin-1β; ICAM-1, intercellular adhesion molecule 1; IL-6, interleukin-6; TNF-α, tumor necrosis factor-α; -S, -sense; -A, -anti-sense.

**Table S2. Description of metabolites quantified CLT_PR vs CLT_EP in this study.**

| **Metabolite** | **VIP** | ***P*-VALUE** | **FOLD CHANGE** | **LOG_FOLD CHANGE** |
| --- | --- | --- | --- | --- |
| Hydroxyacetone | 1.387947032 | 0.027545071 | 1.380426947 | 0.465114542 |
| 1-Aminocyclopropanecarboxylic acid | 1.920527501 | 0.005875218 | 3.527005956 | 1.818444012 |
| Pectin (Galacturonic acid) | 1.338347991 | 0.03694292 | 0.784503954 | -0.350147377 |
| Pyruvaldehyde | 1.167755015 | 0.031162074 | 2.708745046 | 1.43762461 |
| N6-methyladenosine | 2.3136803 | 7.44375E-05 | 0.309915265 | -1.690054278 |
| Guanosine | 1.413564696 | 0.02729504 | 0.514569808 | -0.958561286 |
| 3-Methyluridine | 1.251468701 | 0.003059813 | 3.077040498 | 1.621543431 |
| Taurochenodeoxycholate | 1.873897793 | 0.042939111 | 4.120160719 | 2.042700615 |
| Adenosine | 1.518240033 | 0.003446065 | 1.309915778 | 0.389474056 |
| Inosine | 1.598528969 | 0.011970863 | 0.527360129 | -0.923139593 |
| Ornithine | 1.624841868 | 0.00074568 | 1.622807735 | 0.698492084 |
| O-Phosphotyrosine | 2.187686585 | 0.000113956 | 0.191099753 | -2.387602181 |
| Indole | 2.428836131 | 0.007353738 | 4.631087566 | 2.211351036 |
| 6-Benzylaminopurine | 1.550419693 | 0.013072672 | 3.47483989 | 1.796946505 |
| Cytidine 2',3'-cyclic phosphate | 1.732692081 | 0.002753056 | 0.526782121 | -0.924721715 |
| D-Proline | 1.666269249 | 0.003608428 | 1.440859078 | 0.526929241 |
| 3-methylcytidine | 1.017060679 | 0.047300544 | 2.487708771 | 1.314817603 |
| Cytidine | 1.392864059 | 0.025625909 | 0.514628257 | -0.958397422 |
| Thymine | 1.558883911 | 0.007974777 | 2.414851228 | 1.271934312 |
| Adenylsuccinic acid | 2.129196144 | 0.004743441 | 0.220243979 | -2.182825516 |
| N-Acetyl-L-phenylalanine | 1.787369052 | 0.043909155 | 10.95912011 | 3.454060066 |
| D-Pipecolinic acid | 1.518485133 | 0.001125113 | 1.477625651 | 0.563280816 |
| Uridine 5'-diphosphate (UDP) | 2.0373628 | 0.004865311 | 0.054583522 | -4.195390706 |
| 4-Imidazoleacetic acid | 1.041407633 | 0.046084983 | 0.301592682 | -1.729326672 |
| Uridine 5'-monophosphate (UMP) | 1.577922992 | 0.000506916 | 0.316881636 | -1.657984039 |
| Uridine | 1.547641213 | 0.009860303 | 0.630382496 | -0.665700619 |
| UDP-N-acetylglucosamine | 2.013292499 | 0.011440698 | 0.162879354 | -2.618124353 |
| L-Phenylalanine | 1.060171369 | 0.011756327 | 1.304144788 | 0.383104049 |
| Acetylglycine | 1.802830948 | 0.002165022 | 8.967922787 | 3.164773857 |
| Creatinine | 1.085181229 | 0.031452888 | 1.265714294 | 0.339951786 |
| Methyl acetoacetate | 1.778785706 | 0.024608597 | 0.291063611 | -1.780593612 |
| L-Kynurenine | 1.080427631 | 0.026413297 | 1.770394248 | 0.824070669 |
| Ile-Cys | 1.492787353 | 0.006443276 | 0.424728074 | -1.235388623 |
| N6-Acetyl-L-lysine | 1.378921332 | 0.000620282 | 2.177439139 | 1.122632395 |
| His-Phe | 2.018654401 | 0.008760282 | 0.238390869 | -2.068599116 |
| Ile-Phe | 2.209560391 | 0.005663697 | 0.265513977 | -1.913140284 |
| Acetyl-DL-Leucine | 1.646599203 | 0.024670213 | 2.056458636 | 1.040162054 |
| Val-Val | 1.521253915 | 0.00916536 | 0.40953258 | -1.287949867 |
| Hypoxanthine | 1.616580733 | 0.006158523 | 0.619210764 | -0.691497544 |
| Phloretin | 1.147375219 | 0.003959972 | 3.481101835 | 1.799544019 |
| DL-Serine | 1.83118499 | 0.008966029 | 1.797290536 | 0.845823643 |
| D-Quinovose | 2.174017636 | 0.026826344 | 4.325759492 | 2.112953455 |
| GDP-L-Fucose | 1.765927682 | 0.005075926 | 0.510044015 | -0.971306342 |
| L-Rhamnose | 1.918470515 | 4.00E-08 | 0.369704291 | -1.435556305 |
| Dodecanoic acid | 1.516364301 | 0.002732773 | 1.58098733 | 0.660825806 |
| sn-Glycerol 3-phosphoethanolamine | 1.496421471 | 0.031311783 | 0.646768909 | -0.628677767 |
| 2-Deoxyribose 5-phosphate | 1.643601544 | 0.011741554 | 4.579070817 | 2.195054877 |
| Capric acid | 2.158125242 | 0.004200155 | 2.428920764 | 1.280315428 |
| L-Citrulline | 2.108242918 | 0.012739322 | 4.917630077 | 2.297963214 |
| 3'-O-methyluridine | 1.006070887 | 0.00249056 | 4.684830265 | 2.227996779 |
| D-Maltose | 2.333343493 | 0.00134075 | 0.147925851 | -2.7570539 |
| Betaine | 2.279882253 | 0.000462945 | 2.051354929 | 1.036577131 |
| His-Pro | 1.152030499 | 0.013496842 | 5.203458323 | 2.379470786 |
| D-Threitol | 2.402998621 | 2.07327E-05 | 0.193719719 | -2.367957282 |
| Cytidine 5'-diphosphocholine (CDP-choline) | 1.558603738 | 0.036817788 | 0.483738144 | -1.047701793 |
| Galactonic acid | 1.487543182 | 0.020221317 | 0.659155126 | -0.601310065 |
| L-Histidinol | 1.533788033 | 0.000456557 | 17.86625764 | 4.159165566 |
| Guanosine 5'-diphosphate (GDP) | 1.851637296 | 0.008365876 | 0.498450812 | -1.004476951 |
| His-Val | 1.510781199 | 0.022259381 | 0.299044038 | -1.741570141 |
| N-Carboxyethyl-.gamma.-aminobutyric acid | 1.507814238 | 0.006041924 | 1.901216448 | 0.926922788 |
| S-Nitroso-L-glutathione | 2.195968506 | 0.021617592 | 7.788645814 | 2.961372513 |
| Lisinopril | 1.626886303 | 0.038881052 | 0.553446809 | -0.853483427 |
| L-Valine | 1.849797136 | 0.004149037 | 3.269897758 | 1.709245527 |
| L-Leucine | 1.849802895 | 0.008523214 | 2.008211665 | 1.005911337 |
| Histamine | 1.005530633 | 0.032737007 | 0.287534032 | -1.798195376 |
| Pro-Gln | 2.082716726 | 0.008889986 | 0.297492699 | -1.749073835 |
| Pyridoxamine 5'-phosphate | 1.399926133 | 0.021330238 | 0.460697788 | -1.118107424 |
| Guanosine diphosphate mannose | 2.057988509 | 0.001237089 | 0.211634082 | -2.240356117 |
| Tyr-Gly | 1.629988796 | 0.028845922 | 0.319239328 | -1.647289701 |
| Pyridoxine | 1.39827075 | 0.007133348 | 5.558934601 | 2.474808409 |
| L-Gulonic gamma-lactone | 2.309851955 | 8.8553E-05 | 0.501958165 | -0.994360965 |
| Acamprosate | 1.416870503 | 0.016941527 | 2.541151046 | 1.345482132 |
| L-Galactono-1,4-lactone | 2.891413617 | 6.16225E-05 | 10.92501261 | 3.44956304 |
| Uridine diphosphate glucose(UDP-D-Glucose) | 2.434258404 | 0.011025766 | 0.0697522 | -3.841617473 |
| 3-Phenylpropanoic acid | 2.387867467 | 8.02609E-05 | 13.41070371 | 3.745313037 |
| DL-Methionine sulfoxide | 1.781153948 | 0.001806911 | 2.087058977 | 1.061471369 |
| Nicotinate D-ribonucleotide | 1.564829649 | 0.00037549 | 0.352730642 | -1.503361186 |
| L-Aspartate | 1.30528656 | 0.000604123 | 1.980575653 | 0.985919809 |
| 3-Phosphoserine | 1.591353294 | 0.015042857 | 6.518228553 | 2.704479939 |
| Gamma-Glutamylcysteine | 1.377624591 | 0.012992967 | 0.657904699 | -0.604049478 |
| Adenosine 2',3'-cyclic monophosphate | 2.002715827 | 0.00040134 | 0.334632178 | -1.579351915 |
| N-Phenylacetamide | 1.609682097 | 0.003268538 | 3.66946408 | 1.875569375 |
| Chlorpromazine | 1.908453193 | 0.007671114 | 4.03299028 | 2.011849929 |
| N-Acetyl-L-glutamate | 1.148720198 | 0.021105924 | 1.973526309 | 0.980775752 |
| Psychosine | 1.122832614 | 0.023984599 | 1.556466498 | 0.638274524 |
| Glu-Cys | 1.684574289 | 0.001831678 | 0.562443149 | -0.830220817 |
| Pseudouridine | 1.938351742 | 0.01070748 | 1.85920306 | 0.894684348 |
| Sucrose | 1.969286686 | 0.001635688 | 2.923238548 | 1.547567563 |
| Ser-Asp | 1.054695898 | 0.009425496 | 2.087471318 | 1.061756375 |
| Indolelactic acid | 1.976434633 | 0.003310804 | 2.406292654 | 1.266812114 |
| Isopentenyladenosine | 1.175080692 | 0.019259863 | 1.737964043 | 0.797398234 |
| N-Acetyl-D-lactosamine | 1.220551066 | 0.008113842 | 1.746317179 | 0.804315615 |
| L-Iditol | 1.661564709 | 6.44546E-08 | 0.386351575 | -1.372013815 |
| 5-Aminopentanoic acid | 1.982500032 | 0.006704374 | 0.389158369 | -1.361570712 |
| 3-Deoxy-2-keto-6-phosphogluconic acid | 1.732007098 | 0.020101564 | 0.405844078 | -1.301002533 |
| Theobromine | 2.314668893 | 0.00051406 | 7.027675535 | 2.813047584 |
| L-Cystine | 1.592784893 | 0.046522422 | 0.378416836 | -1.40195182 |
| Glu-Pro | 1.182721288 | 0.036150731 | 1.785016076 | 0.835937068 |
| Arg-Thr | 1.66019013 | 0.012963095 | 0.451364535 | -1.14763503 |
| 2-Hydroxyadenine | 1.407253327 | 0.041925306 | 0.529820224 | -0.916425181 |
| Glyceric acid | 1.156196374 | 0.001942398 | 1.824864133 | 0.867789055 |
| Thr-Glu | 1.54892061 | 0.009996693 | 0.739642245 | -0.435100468 |
| Thymidine | 2.18098186 | 5.62919E-07 | 0.464188692 | -1.107216716 |
| Ala-Val | 1.45189435 | 0.049787353 | 3.867745002 | 1.951492682 |
| N-carbamoyl-L-aspartate | 1.593201815 | 0.001869388 | 2.210905704 | 1.144637495 |
| Met-His | 1.562808761 | 0.002044948 | 0.482126815 | -1.052515423 |
| Equol | 1.576304225 | 0.02733163 | 0.324063544 | -1.625651365 |
| 2-Dehydro-3-deoxy-D-gluconate | 1.900335409 | 0.000316223 | 0.358351936 | -1.480550948 |
| Altretamine | 1.334387724 | 0.029094221 | 0.741224549 | -0.432017432 |
| Phe-Trp | 1.965967438 | 0.046477871 | 0.370704009 | -1.431660378 |
| D-Glucono-1,5-lactone | 1.409510719 | 3.34481E-07 | 0.347252486 | -1.525943071 |
| 2-Methyl-3-hydroxybutyric acid | 1.329833404 | 0.009300624 | 1.657850812 | 0.729314186 |
| 3-Aminosalicylic acid | 1.630381911 | 0.042405798 | 0.136778968 | -2.870081689 |
| O-Succinyl-L-homoserine | 1.001165745 | 0.030664526 | 1.447296491 | 0.5333605 |
| Glutathione | 1.850689005 | 0.000285598 | 3.849122633 | 1.944529636 |
| 5-Methylcytosine | 2.113959432 | 0.013916595 | 6.71198663 | 2.746739842 |
| DL-Indole-3-lactic acid | 2.709695266 | 2.87037E-08 | 0.126184585 | -2.986392421 |
| Phenylethylamine | 1.524600129 | 0.008470617 | 2.08141692 | 1.057565974 |
| Hydrocortisone | 1.342071566 | 0.011411814 | 0.419441489 | -1.253458524 |
| Ile-Ala-Arg | 1.521002118 | 0.004022446 | 2.479263932 | 1.309911863 |
| Met-Thr | 1.246465067 | 0.00267911 | 4.385174373 | 2.132634211 |
| Glu-His | 1.231372071 | 0.040566172 | 0.701620123 | -0.511237968 |
| Estrone-3-glucuronide | 1.14603552 | 0.004823803 | 3.572277406 | 1.836844117 |
| DL-O-tyrosine | 1.950634298 | 0.001368476 | 1.897448449 | 0.92406069 |
| 2-Oleoyl-1-stearoyl-sn-glycero-3-phosphoserine | 1.020463099 | 0.034448705 | 2.364437787 | 1.241497182 |
| Phe-Asn | 1.958858835 | 0.009953472 | 1.60706398 | 0.684427366 |
| L-Arabinono-1,4-lactone | 1.912320889 | 0.006710436 | 2.516274409 | 1.331289262 |
| N,N-Bis(2-hydroxyethyl)glycine | 1.614384012 | 0.001183952 | 2.035620447 | 1.025468588 |
| Asp-Arg | 1.146073398 | 0.024770447 | 0.382608651 | -1.3860586 |
| His-Trp | 1.894925133 | 0.000217163 | 0.33427129 | -1.580908647 |
| Prostaglandin E2 | 1.20215729 | 0.030085535 | 3.30743578 | 1.725713144 |
| Isocaproic acid | 2.086136892 | 0.000284134 | 97.08264304 | 6.601141481 |
| 1-Oleoyl-sn-glycerol 3-phosphate | 1.219149769 | 0.020364161 | 0.586814678 | -0.769023136 |
| Isopentenyl pyrophosphate | 1.481846989 | 0.028516283 | 0.547841715 | -0.868168973 |
| N-Formylmethionine | 2.187117672 | 0.005257671 | 4.042788021 | 2.01535056 |
| Serotonin | 1.440106261 | 0.001536683 | 1.573463708 | 0.653943903 |
| Meclofenamate | 1.672979417 | 0.002851361 | 2.633022785 | 1.396720006 |
| 2'-O-Methyluridine | 1.685290737 | 0.019502492 | 0.210098189 | -2.25086437 |
| Arg-Gln | 1.481416925 | 0.011520651 | 0.331743453 | -1.591860102 |
| D-Allose | 1.148364054 | 0.010609151 | 1.628540423 | 0.703579531 |
| Alpha.-L-Asp-L-Lys | 1.250317618 | 0.02296029 | 0.247826695 | -2.012596496 |
| Rhapontigenin | 2.003892146 | 0.002510303 | 0.507005289 | -0.979927297 |
| 2'-Deoxy-D-ribose | 2.453154302 | 0.001372548 | 5.136990025 | 2.360923272 |
| Synephrine | 2.331067224 | 3.78856E-06 | 6.450431459 | 2.689395663 |
| Ethylmalonic acid | 2.351903935 | 2.68E-07 | 0.356085296 | -1.489705231 |
| trans-cinnamate | 1.45439857 | 0.031380762 | 1.989698221 | 0.992549633 |

CLT, conventional liver transplantation; EP, end-preservation; PR, postrevascularization; VIP, variable importance in the projection.

**Table S3. Description of metabolites quantified IFLT_PR vs IFLT_EP in this study.**

| **Metabolite** | **VIP** | **P-VALUE** | **FOLD CHANGE** | **LOG_FOLD CHANGE** |
| --- | --- | --- | --- | --- |
| N2-Acetyl-L-ornithine | 1.394279096 | 0.028625942 | 0.644581005 | -0.63356642 |
| Glycocholic acid | 1.540991086 | 0.049727028 | 1.670389408 | 0.740184469 |
| Oleoyl-CoA | 2.572241821 | 0.00071915 | 6.198435179 | 2.631904047 |
| Pro-Phe | 1.366875828 | 0.016877545 | 0.512119269 | -0.965448252 |
| Adenosine | 1.516138856 | 0.009724417 | 0.778877096 | -0.360532401 |
| Taurocholate | 2.151449837 | 0.00357818 | 2.296629925 | 1.199518402 |
| O-Phosphotyrosine | 2.541225436 | 2.89174E-05 | 0.27564798 | -1.859101067 |
| L-Pipecolic acid | 1.541867203 | 0.00350747 | 0.475060871 | -1.073815712 |
| Glycodeoxycholic acid | 1.766870723 | 0.004491643 | 3.399114425 | 1.765158928 |
| Kynurenic acid | 1.988885867 | 0.001423003 | 0.240040734 | -2.058648847 |
| 4-Imidazoleacetic acid | 1.846486873 | 0.001742177 | 1.809165753 | 0.855324592 |
| 4-Pyridoxic acid | 1.591737565 | 0.015849393 | 1.481661507 | 0.567215894 |
| all cis-(6,9,12)-Linolenic acid | 1.309692426 | 0.04462879 | 1.599401454 | 0.677532104 |
| N-Acetylneuraminic acid | 1.518252622 | 0.041818254 | 1.293900976 | 0.37172721 |
| Raffinose | 2.543044185 | 8.59204E-05 | 2.095751795 | 1.067467865 |
| gamma-L-Glutamyl-L-glutamic acid | 1.895762281 | 0.007428686 | 1.812129784 | 0.857686284 |
| 5-methoxyuridine | 1.312083863 | 0.023094784 | 0.586909986 | -0.76878884 |
| Phloretin | 1.502498741 | 0.019640881 | 1.774409338 | 0.827338863 |
| Urocanic acid | 1.906107375 | 0.003842316 | 2.705584797 | 1.435940458 |
| His-Gly | 1.363758307 | 0.008612547 | 0.499234442 | -1.002210626 |
| Phosphorylcholine | 1.363759997 | 0.021416762 | 1.276892552 | 0.352637131 |
| His-Glu | 1.371189112 | 0.007377555 | 0.465607198 | -1.102814733 |
| D-Quinovose | 2.114745278 | 0.045065317 | 3.771251866 | 1.915043505 |
| Ser-Ala | 1.293743681 | 0.047916195 | 0.569318134 | -0.812693041 |
| L-Rhamnose | 3.004277178 | 1.79E-09 | 0.447689134 | -1.159430792 |
| Dodecanoic acid | 1.675219692 | 0.011399679 | 1.518672323 | 0.60281062 |
| Capric acid | 2.103583618 | 0.004395388 | 2.336328005 | 1.224242833 |
| NG,NG-dimethyl-L-arginine(ADMA) | 1.575092307 | 0.002599122 | 1.613214078 | 0.689937901 |
| D-Maltose | 1.930220481 | 0.000490617 | 0.380894768 | -1.392535624 |
| L-Threonate | 1.386566782 | 0.007177428 | 0.641632972 | -0.640179813 |
| 16-Hydroxypalmitic acid | 1.775197788 | 0.041979423 | 1.453072666 | 0.539106852 |
| His-Pro | 2.640662261 | 0.000665464 | 0.316898749 | -1.65790613 |
| D-Threitol | 2.813438485 | 4.21E-06 | 0.358283776 | -1.480825379 |
| Indoxyl sulfate | 1.378545009 | 0.02132217 | 2.473448573 | 1.306523904 |
| Hippuric acid | 1.98478481 | 0.00104178 | 0.212975927 | -2.231237723 |
| Galactonic acid | 2.172669551 | 0.000260984 | 0.660346359 | -0.598705163 |
| L-Histidinol | 3.176643591 | 6.21916E-07 | 19.61902064 | 4.294181121 |
| Xylitol | 1.767959283 | 0.009891968 | 0.472000075 | -1.083141007 |
| N-Carboxyethyl-.gamma.-aminobutyric acid | 1.796418007 | 0.015518918 | 0.525220319 | -0.929005365 |
| Pyridoxine | 2.273228914 | 2.21758E-05 | 0.386402658 | -1.371823076 |
| L-Gulonic gamma-lactone | 1.65277446 | 0.004192792 | 0.667975192 | -0.582133572 |
| Pro-Thr | 1.104861648 | 0.026689704 | 1.719174996 | 0.781716406 |
| L-NG-Monomethylarginine | 1.445124424 | 0.004018488 | 1.444837891 | 0.530907633 |
| L-Galactono-1,4-lactone | 2.82602484 | 6.59E-06 | 8.747600436 | 3.128887324 |
| 3-Hydroxybenzoate | 1.482062345 | 0.036395343 | 1.201577805 | 0.26493007 |
| 3-Phenylpropanoic acid | 2.367634323 | 0.00059887 | 3.714498191 | 1.893167323 |
| Tauroursodeoxycholic acid | 1.964583557 | 0.006716933 | 2.20321504 | 1.139610313 |
| Tetrahydrobiopterin | 1.248347687 | 0.023090106 | 0.555082341 | -0.849226299 |
| 3-Hydroxyisovaleric acid | 2.562288361 | 0.000140061 | 0.371851059 | -1.427203215 |
| Farnesyl pyrophosphate | 1.765881231 | 0.02219076 | 0.501671852 | -0.995184103 |
| Tyramine | 1.798700273 | 0.030357167 | 1.250354792 | 0.322337523 |
| 6.alpha.-Methylprednisolone | 1.280650873 | 0.005321122 | 0.185017004 | -2.434270226 |
| Adenosine 2',3'-cyclic monophosphate | 1.824732906 | 0.003003923 | 0.647551827 | -0.62693243 |
| 3-Methoxytyramine | 1.527831346 | 0.040137516 | 4.024393158 | 2.008771254 |
| Donepezil | 1.452217719 | 0.015023989 | 3.195002144 | 1.675816899 |
| Chlorpromazine | 1.827183534 | 0.026220426 | 0.494124824 | -1.017052558 |
| .alpha.-L-Glu-L-Asp | 1.778344747 | 0.003834696 | 0.31382101 | -1.671986153 |
| Ser-Asp | 1.574799301 | 0.020687459 | 1.540794332 | 0.623674301 |
| Aspartame | 1.416778177 | 0.023732741 | 0.532640031 | -0.908767234 |
| L-Iditol | 3.010545726 | 5.09558E-11 | 0.479684265 | -1.059842978 |
| Maltotriose | 2.091199593 | 6.34241E-05 | 2.309534999 | 1.207602409 |
| Gly-Glu | 1.505144429 | 0.041169784 | 1.539460158 | 0.62242453 |
| 3,5-Dibromo-L-tyrosine | 1.720566673 | 0.044249199 | 1.399021864 | 0.484418509 |
| 1-Methylnicotinamide | 1.10513918 | 0.044703635 | 1.283650577 | 0.360252539 |
| D-Galactarate | 1.626114935 | 0.001392916 | 0.43235185 | -1.209722231 |
| Diacetyl | 1.752177788 | 0.000872345 | 0.484217821 | -1.046271917 |
| Diacetyl | 1.46974322 | 0.038237714 | 0.588897908 | -0.763910546 |
| Trp-Trp | 1.308733387 | 0.005012316 | 0.115671962 | -3.111888883 |
| Thymidine | 2.107126349 | 0.000764902 | 0.634794779 | -0.655637832 |
| 3-Methoxy-4-Hydroxyphenylglycol Sulfate | 2.238083344 | 0.001601039 | 3.835691218 | 1.939486585 |
| Monomethyl glutaric acid | 1.13596917 | 0.032576812 | 0.483550668 | -1.048261026 |
| DL-Indole-3-lactic acid | 2.902968233 | 6.51833E-07 | 0.257328663 | -1.958315934 |
| Cellobiose | 1.268383639 | 0.049758864 | 0.627040276 | -0.673369981 |
| Phe-Cys | 1.500953754 | 0.023309409 | 0.579651896 | -0.786741331 |
| Heptadecanoic acid | 1.664744759 | 0.016171826 | 1.937908113 | 0.954500166 |
| 5-Hydroxytryptophol (5HTOL) | 2.308057132 | 0.000822886 | 0.609413162 | -0.714507435 |
| Ethanolamine | 1.785119303 | 0.00113515 | 2.075884379 | 1.053726092 |
| Isocaproic acid | 3.0378696 | 4.11E-07 | 19.41104163 | 4.278805632 |
| Trp-Cys | 1.197622208 | 0.025631585 | 0.188197801 | -2.409678323 |
| Hydroxyproline | 1.006586406 | 0.024315518 | 3.506120393 | 1.809875536 |
| -Benzenetriol | 1.731879892 | 0.016396649 | 1.619859397 | 0.695868593 |
| D-Allose | 1.358918929 | 0.037599982 | 1.346369045 | 0.429073912 |
| 3-Furancarboxylic acid | 1.404878928 | 0.025226342 | 1.21442307 | 0.280271102 |
| Beta-Alanine | 1.178206754 | 0.008639156 | 0.563102744 | -0.828529915 |

IFLT, ischemia-free liver transplantation; EP, end-preservation; PR, postrevascularization; VIP, variable importance in the projection.

| **Class** | **Nucleosides, nucleotides and derivatives** | **Amino acid and derivatives** | **Lipid** | **Carbohydrate** | **Vitamins** | **Hormone** | **Fatty acid** | **Organic acid**  **(Carboxylic acid)** |
| --- | --- | --- | --- | --- | --- | --- | --- | --- |
| CLT_PR vs CLT_EP  (lower) | 18 | 3 | 4 | 3 | 0 | 0 | 0 | 6 |
| CLT_PR vs CLT_EP  (higher) | 6 | 14 | 2 | 4 | 1 | 1 | 3 | 17 |
| IFLT_PR vs IFLT_EP  (lower) | 4 | 3 | 3 | 5 | 0 | 0 | 0 | 4 |
| IFLT_PR vs IFLT_EP  (higher) | 0 | 2 | 2 | 4 | 1 | 0 | 5 | 4 |

**Table S4. The metabolite classification of CLT_PR vs CLT_EP and IFLT_PR vs IFLT_EP**.

(PR vs. EP), post-revascularization versus end of preservation; IFLT, ischemia-free liver transplantation; CLT, conventional liver transplantation.

**Table S5. Top 10 differentially expressed genes (DEGs) with the smallest *P* values after graft re-vascularization．**

| Rank | Gene Symbol | | Gene Description | p-value | log2(FC) |
| --- | --- | --- | --- | --- | --- |
| **IFLT (PR vs. EP)** | | | |  |  |
| 1 | ST6GAL2 | ST6 beta-galactoside alpha-2,6-sialyltransferase 2 | | 1.09E-10 | -1.9 |
| 2 | SLC7A11 | solute carrier family 7 member 11 | | 1.46E-08 | 3.0 |
| 3 | ZBTB43 | zinc finger and BTB domain containing 43 | | 1.88E-08 | 0.8 |
| 4 | CLDN5 | claudin 5 | | 2.39E-08 | -1.2 |
| 5 | KLF8 | Kruppel like factor 8 | | 4.03E-08 | -1.6 |
| 6 | RNF139 | ring finger protein 139 | | 6.08E-08 | 0.4 |
| 7 | SASH1 | SAM and SH3 domain containing 1 | | 7.02E-08 | 0.4 |
| 8 | POLR3C | RNA polymerase III subunit C | | 7.29E-08 | 0.7 |
| 9 | ARHGEF17 | Rho guanine nucleotide exchange factor 17 | | 9.33E-08 | -0.9 |
| 10 | REM1 | RRAD and GEM like GTPase 1 | | 1.19E-07 | -2.2 |
| **CLT (PR vs. EP)** | | | |  |  |
| 1 | HSPA1A | heat shock protein family A (Hsp70) member 1A | | 1.00E-32 | 5.0 |
| 2 | DNAJB1 | DnaJ heat shock protein family (Hsp40) member B1 | | 7.07E-30 | 3.9 |
| 3 | DNAJA4 | DnaJ heat shock protein family (Hsp40) member A4 | | 3.42E-28 | 4.3 |
| 4 | FOSL1 | FOS like 1, AP-1 transcription factor subunit | | 6.95E-27 | 6.1 |
| 5 | ATF3 | activating transcription factor 3 | | 1.45E-26 | 4.2 |
| 6 | HSPA6 | heat shock protein family A (Hsp70) member 6 | | 4.60E-26 | 5.8 |
| 7 | TRIB1 | tribbles pseudokinase 1 | | 6.16E-26 | 2.9 |
| 8 | NR4A3 | nuclear receptor subfamily 4 group A member 3 | | 7.40E-26 | 5.9 |
| 9 | BAG3 | BCL2 associated athanogene 3 | | 9.27E-25 | 4.1 |
| 10 | MXD1 | MAX dimerization protein 1 | | 1.80E-24 | 2.7 |

(PR vs. EP), postrevascularization versus end-preservation; IFLT, ischemia-free liver transplantation; CLT, conventional liver transplantation; FC, fold change.

**Table S6. Enzymes and enzyme-related proteins in differential proteins in the IFLT_EP versus CLT_EP groups.**

| UniProt ID | Protein Name | Protein Description | P Value | Fold Change |
| --- | --- | --- | --- | --- |
| Q6P1A2 | MBOA5 | Lysophospholipid acyltransferase 5 | 7.52E-09 | 5.61 |
| Q96N66 | MBOA7 | Lysophospholipid acyltransferase 7 | 1.08E-09 | 5.34 |
| Q2PZI1 | D19L1 | Probable C-mannosyltransferase DPY19L1 | 2.41E-09 | 5.11 |
| P00915 | CAH1 | Carbonic anhydrase 1 | 4.10E-13 | 4.64 |
| Q8TDZ2 | MICA1 | [F-actin]-monooxygenase MICAL1 | 6.49E-16 | 4.64 |
| P07738 | PMGE | Bisphosphoglycerate mutase | 1.53E-11 | 4.48 |
| Q9ULK4 | MED23 | Mediator of RNA polymerase II transcription subunit 23 | 1.69E-16 | 4.36 |
| Q8NB49 | AT11C | Phospholipid-transporting ATPase IG | 1.52E-08 | 4.35 |
| O75319 | DUS11 | RNA/RNP complex-1-interacting phosphatase | 6.05E-07 | 4.32 |
| Q9Y6K0 | CEPT1 | Choline/ethanolaminephosphotransferase 1 | 7.04E-09 | 4.29 |
| P14780 | MMP9 | Matrix metalloproteinase-9 | 3.05E-05 | 4.25 |
| P42357 | HUTH | Histidine ammonia-lyase | 6.91E-12 | 4.23 |
| Q8N8Q8 | COX18 | Cytochrome c oxidase assembly protein COX18, mitochondrial | 1.03E-08 | 4.21 |
| P03905 | NU4M | NADH-ubiquinone oxidoreductase chain 4 | 2.39E-08 | 3.95 |
| Q9H6U8 | ALG9 | Alpha-1,2-mannosyltransferase ALG9 | 4.19E-05 | 3.90 |
| O75116 | ROCK2 | Rho-associated protein kinase 2 | 2.12E-07 | 3.83 |
| P50993 | AT1A2 | Sodium/potassium-transporting ATPase subunit alpha-2 | 1.89E-07 | 3.80 |
| P78540 | ARGI2 | Arginase-2, mitochondrial | 7.52E-04 | 3.79 |
| Q93050 | VPP1 | V-type proton ATPase 116 kDa subunit a isoform 1 | 1.13E-08 | 3.78 |
| Q9P035 | HACD3 | Very-long-chain (3R)-3-hydroxyacyl-CoA dehydratase 3 | 2.35E-08 | 3.77 |
| O75907 | DGAT1 | Diacylglycerol O-acyltransferase 1 | 1.81E-07 | 3.72 |
| Q9Y672 | ALG6 | Dolichyl pyrophosphate Man9GlcNAc2 alpha-1,3-glucosyltransferase | 7.28E-09 | 3.70 |
| O14732 | IMPA2 | Inositol monophosphatase 2 | 7.51E-09 | 3.68 |
| P46977 | STT3A | Dolichyl-diphosphooligosaccharide--protein glycosyltransferase subunit STT3A | 3.00E-08 | 3.66 |
| P00414 | COX3 | Cytochrome c oxidase subunit 3 | 5.32E-09 | 3.60 |
| Q92685 | ALG3 | Dol-P-Man:Man(5)GlcNAc(2)-PP-Dol alpha-1,3-mannosyltransferase | 1.35E-07 | 3.52 |
| Q9HD20 | AT131 | Manganese-transporting ATPase 13A1 | 1.35E-09 | 3.50 |
| P08183 | MDR1 | ATP-dependent translocase ABCB1 | 3.60E-08 | 3.41 |
| Q8WU67 | ABHD3 | Phospholipase ABHD3 | 1.38E-04 | 3.39 |
| P48651 | PTSS1 | Phosphatidylserine synthase 1 | 5.78E-08 | 3.38 |
| Q6ZNB7 | ALKMO | Alkylglycerol monooxygenase | 1.72E-08 | 3.36 |
| Q7Z3V4 | UBE3B | Ubiquitin-protein ligase E3B | 4.79E-03 | 3.34 |
| Q9H0A0 | NAT10 | RNA cytidine acetyltransferase | 1.18E-04 | 3.32 |
| Q9UBM7 | DHCR7 | 7-dehydrocholesterol reductase | 6.34E-07 | 3.31 |
| Q8TCJ2 | STT3B | Dolichyl-diphosphooligosaccharide--protein glycosyltransferase subunit STT3B | 2.00E-08 | 3.31 |
| P42336 | PK3CA | Phosphatidylinositol 4,5-bisphosphate 3-kinase catalytic subunit alpha isoform | 2.20E-09 | 3.28 |
| P56192 | SYMC | Methionine--tRNA ligase, cytoplasmic | 1.48E-09 | 3.28 |
| Q9NRZ7 | PLCC | 1-acyl-sn-glycerol-3-phosphate acyltransferase gamma | 1.33E-07 | 3.27 |
| Q14397 | GCKR | Glucokinase regulatory protein | 1.23E-07 | 3.25 |
| Q86X10 | RLGPB | Ral GTPase-activating protein subunit beta | 7.32E-10 | 3.24 |
| Q6ZMG9 | CERS6 | Ceramide synthase 6 | 8.49E-07 | 3.19 |
| P33121 | ACSL1 | Long-chain-fatty-acid--CoA ligase 1 | 3.83E-08 | 3.17 |
| Q9BVK2 | ALG8 | Probable dolichyl pyrophosphate Glc1Man9GlcNAc2 alpha-1,3-glucosyltransferase | 2.44E-09 | 3.16 |
| Q7Z2K6 | ERMP1 | Endoplasmic reticulum metallopeptidase 1 | 4.86E-04 | 3.15 |
| O14756 | H17B6 | 17-beta-hydroxysteroid dehydrogenase type 6 | 6.58E-06 | 3.12 |
| Q9Y2P5 | S27A5 | Bile acyl-CoA synthetase | 2.54E-06 | 3.09 |
| O76062 | ERG24 | Delta(14)-sterol reductase TM7SF2 | 2.03E-08 | 3.09 |
| Q9H3H5 | GPT | UDP-N-acetylglucosamine--dolichyl-phosphate N-acetylglucosaminephosphotransferase | 2.36E-08 | 3.08 |
| O14920 | IKKB | Inhibitor of nuclear factor kappa-B kinase subunit beta | 5.11E-11 | 3.07 |
| P49327 | FAS | Fatty acid synthase | 1.34E-06 | 3.06 |
| P07099 | HYEP | Epoxide hydrolase 1 | 2.77E-06 | 3.05 |
| Q9NZ01 | TECR | Very-long-chain enoyl-CoA reductase | 1.02E-09 | 3.02 |
| O95352 | ATG7 | Ubiquitin-like modifier-activating enzyme ATG7 | 1.25E-09 | 3.00 |
| O75643 | U520 | U5 small nuclear ribonucleoprotein 200 kDa helicase | 4.51E-07 | 2.99 |
| O75844 | FACE1 | CAAX prenyl protease 1 homolog | 7.83E-08 | 2.98 |
| Q15124 | PGM5 | Phosphoglucomutase-like protein 5 | 3.20E-11 | 2.94 |
| Q8NBQ5 | DHB11 | Estradiol 17-beta-dehydrogenase 11 | 4.97E-08 | 2.94 |
| Q13423 | NNTM | NAD(P) transhydrogenase, mitochondrial | 1.88E-09 | 2.93 |
| O14772 | FPGT | Fucose-1-phosphate guanylyltransferase | 1.74E-07 | 2.93 |
| Q9NTJ5 | SAC1 | Phosphatidylinositide phosphatase SAC1 | 2.94E-09 | 2.92 |
| P53609 | PGTB1 | Geranylgeranyl transferase type-1 subunit beta | 1.58E-12 | 2.92 |
| Q8WUD6 | CHPT1 | Cholinephosphotransferase 1 | 4.10E-09 | 2.91 |
| O00519 | FAAH1 | Fatty-acid amide hydrolase 1 | 2.10E-07 | 2.91 |
| Q92696 | PGTA | Geranylgeranyl transferase type-2 subunit alpha | 8.48E-10 | 2.91 |
| Q9UBM1 | PEMT | Phosphatidylethanolamine N-methyltransferase | 2.79E-07 | 2.90 |
| P36222 | CH3L1 | Chitinase-3-like protein 1 | 1.42E-05 | 2.89 |
| Q2TAA5 | ALG11 | GDP-Man:Man(3)GlcNAc(2)-PP-Dol alpha-1,2-mannosyltransferase | 1.40E-07 | 2.88 |
| Q8IZV5 | RDH10 | Retinol dehydrogenase 10 | 1.47E-08 | 2.88 |
| P09917 | LOX5 | Arachidonate 5-lipoxygenase | 1.59E-06 | 2.87 |
| P03915 | NU5M | NADH-ubiquinone oxidoreductase chain 5 | 7.15E-09 | 2.87 |
| O43242 | PSMD3 | 26S proteasome non-ATPase regulatory subunit 3 | 6.28E-08 | 2.86 |
| P24557 | THAS | Thromboxane-A synthase | 5.30E-08 | 2.86 |
| Q9NX47 | MARH5 | E3 ubiquitin-protein ligase MARCHF5 | 1.77E-07 | 2.85 |
| P03923 | NU6M | NADH-ubiquinone oxidoreductase chain 6 | 1.41E-08 | 2.85 |
| P46019 | KPB2 | Phosphorylase b kinase regulatory subunit alpha, liver isoform | 5.69E-05 | 2.84 |
| Q9C0D9 | EPT1 | Ethanolaminephosphotransferase 1 | 9.22E-07 | 2.83 |
| P53611 | PGTB2 | Geranylgeranyl transferase type-2 subunit beta | 1.64E-08 | 2.82 |
| P23634 | AT2B4 | Plasma membrane calcium-transporting ATPase 4 | 5.43E-09 | 2.82 |
| P00846 | ATP6 | ATP synthase subunit a | 5.80E-08 | 2.80 |
| P20292 | AL5AP | Arachidonate 5-lipoxygenase-activating protein | 2.05E-05 | 2.77 |
| Q5VWC8 | HACD4 | Very-long-chain (3R)-3-hydroxyacyl-CoA dehydratase 4 | 6.06E-05 | 2.77 |
| Q63HN8 | RN213 | E3 ubiquitin-protein ligase RNF213 | 7.27E-07 | 2.77 |
| O15254 | ACOX3 | Peroxisomal acyl-coenzyme A oxidase 3 | 9.91E-06 | 2.77 |
| Q9NP80 | PLPL8 | Calcium-independent phospholipase A2-gamma | 4.20E-08 | 2.76 |
| Q969N2 | PIGT | GPI transamidase component PIG-T | 2.66E-05 | 2.75 |
| Q9UBV7 | B4GT7 | Beta-1,4-galactosyltransferase 7 | 7.40E-07 | 2.73 |
| Q86TP1 | PRUN1 | Exopolyphosphatase PRUNE1 | 2.86E-07 | 2.73 |
| P00395 | COX1 | Cytochrome c oxidase subunit 1 | 2.91E-08 | 2.71 |
| P26640 | SYVC | Valine--tRNA ligase | 4.05E-10 | 2.69 |
| Q99570 | PI3R4 | Phosphoinositide 3-kinase regulatory subunit 4 | 8.62E-08 | 2.69 |
| Q00722 | PLCB2 | 1-phosphatidylinositol 4,5-bisphosphate phosphodiesterase beta-2 | 5.51E-07 | 2.69 |
| Q7Z5P4 | DHB13 | 17-beta-hydroxysteroid dehydrogenase 13 | 5.15E-04 | 2.68 |
| P30711 | GSTT1 | Glutathione S-transferase theta-1 | 7.39E-06 | 2.68 |
| Q96S06 | LMF1 | Lipase maturation factor 1 | 7.42E-07 | 2.68 |
| Q14739 | LBR | Delta(14)-sterol reductase LBR | 1.27E-08 | 2.68 |
| P05023 | AT1A1 | Sodium/potassium-transporting ATPase subunit alpha-1 | 5.68E-11 | 2.67 |
| Q02108 | GCYA1 | Guanylate cyclase soluble subunit alpha-1 | 4.40E-05 | 2.66 |
| Q709F0 | ACD11 | Acyl-CoA dehydrogenase family member 11 | 3.12E-07 | 2.66 |
| P31513 | FMO3 | Dimethylaniline monooxygenase [N-oxide-forming] 3 | 7.41E-07 | 2.65 |
| Q8IY17 | PLPL6 | Neuropathy target esterase | 2.71E-11 | 2.63 |
| Q8IY21 | DDX60 | Probable ATP-dependent RNA helicase DDX60 | 2.63E-07 | 2.63 |
| P35573 | GDE | Glycogen debranching enzyme | 2.98E-09 | 2.62 |
| O15228 | GNPAT | Dihydroxyacetone phosphate acyltransferase | 5.51E-06 | 2.62 |
| Q9H0K1 | SIK2 | Serine/threonine-protein kinase SIK2 | 1.20E-04 | 2.62 |
| P53396 | ACLY | ATP-citrate synthase | 9.18E-07 | 2.61 |
| P31512 | FMO4 | Dimethylaniline monooxygenase [N-oxide-forming] 4 | 2.26E-09 | 2.61 |
| Q8TD19 | NEK9 | Serine/threonine-protein kinase Nek9 | 1.04E-09 | 2.60 |
| Q9NVE7 | PANK4 | 4'-phosphopantetheine phosphatase | 5.47E-10 | 2.60 |
| P03886 | NU1M | NADH-ubiquinone oxidoreductase chain 1 | 1.62E-05 | 2.59 |
| O60906 | NSMA | Sphingomyelin phosphodiesterase 2 | 1.02E-04 | 2.58 |
| Q9NUQ2 | PLCE | 1-acyl-sn-glycerol-3-phosphate acyltransferase epsilon | 8.78E-09 | 2.57 |
| O95470 | SGPL1 | Sphingosine-1-phosphate lyase 1 | 6.15E-08 | 2.56 |
| O14735 | CDIPT | CDP-diacylglycerol--inositol 3-phosphatidyltransferase | 2.37E-06 | 2.55 |
| Q5H8A4 | PIGG | GPI ethanolamine phosphate transferase 2 | 1.69E-05 | 2.55 |
| P47897 | SYQ | Glutamine--tRNA ligase | 6.73E-10 | 2.55 |
| P78527 | PRKDC | DNA-dependent protein kinase catalytic subunit | 9.34E-08 | 2.55 |
| P46063 | RECQ1 | ATP-dependent DNA helicase Q1 | 9.08E-07 | 2.54 |
| P17735 | ATTY | Tyrosine aminotransferase | 7.43E-08 | 2.54 |
| P18074 | ERCC2 | General transcription and DNA repair factor IIH helicase subunit XPD | 1.07E-05 | 2.54 |
| O00763 | ACACB | Acetyl-CoA carboxylase 2 | 7.67E-09 | 2.54 |
| Q6PCE3 | PGM2L | Glucose 1,6-bisphosphate synthase | 3.02E-08 | 2.54 |
| Q15008 | PSMD6 | 26S proteasome non-ATPase regulatory subunit 6 | 2.01E-08 | 2.53 |
| P50416 | CPT1A | Carnitine O-palmitoyltransferase 1, liver isoform | 1.32E-07 | 2.52 |
| P48449 | ERG7 | Lanosterol synthase | 1.05E-07 | 2.52 |
| P16615 | AT2A2 | Sarcoplasmic/endoplasmic reticulum calcium ATPase 2 | 8.21E-10 | 2.51 |
| O14975 | S27A2 | Very long-chain acyl-CoA synthetase | 1.49E-06 | 2.50 |
| Q9H0J9 | PAR12 | Protein mono-ADP-ribosyltransferase PARP12 | 1.94E-04 | 2.50 |
| O60488 | ACSL4 | Long-chain-fatty-acid--CoA ligase 4 | 1.82E-04 | 2.50 |
| Q8N6H7 | ARFG2 | ADP-ribosylation factor GTPase-activating protein 2 | 3.33E-08 | 2.49 |
| Q8TEQ8 | PIGO | GPI ethanolamine phosphate transferase 3 | 6.00E-03 | 2.49 |
| Q13976 | KGP1 | cGMP-dependent protein kinase 1 | 3.44E-08 | 2.47 |
| Q687X5 | STEA4 | Metalloreductase STEAP4 | 3.47E-07 | 2.47 |
| P15088 | CBPA3 | Mast cell carboxypeptidase A | 1.33E-09 | 2.47 |
| Q15031 | SYLM | Probable leucine--tRNA ligase, mitochondrial | 1.09E-07 | 2.47 |
| Q9UNU6 | CP8B1 | 7-alpha-hydroxycholest-4-en-3-one 12-alpha-hydroxylase | 1.20E-05 | 2.47 |
| Q8IZH2 | XRN1 | 5'-3' exoribonuclease 1 | 3.12E-07 | 2.46 |
| O60306 | AQR | RNA helicase aquarius | 7.55E-08 | 2.46 |
| P52790 | HXK3 | Hexokinase-3 | 5.63E-07 | 2.46 |
| O00767 | ACOD | Acyl-CoA desaturase | 9.13E-03 | 2.45 |
| P52209 | 6PGD | 6-phosphogluconate dehydrogenase, decarboxylating | 2.97E-10 | 2.44 |
| Q15392 | DHC24 | Delta(24)-sterol reductase | 2.41E-07 | 2.44 |
| Q9UPQ8 | DOLK | Dolichol kinase | 2.63E-04 | 2.44 |
| P10398 | ARAF | Serine/threonine-protein kinase A-Raf | 3.76E-11 | 2.43 |
| O15229 | KMO | Kynurenine 3-monooxygenase | 7.67E-07 | 2.43 |
| Q6UX53 | MET7B | Methyltransferase-like protein 7B | 3.38E-09 | 2.41 |
| P07451 | CAH3 | Carbonic anhydrase 3 | 1.69E-03 | 2.40 |
| P13807 | GYS1 | Glycogen [starch] synthase, muscle | 3.48E-07 | 2.40 |
| Q8N8A2 | ANR44 | Serine/threonine-protein phosphatase 6 regulatory ankyrin repeat subunit B | 1.53E-06 | 2.40 |
| Q9BW92 | SYTM | Threonine--tRNA ligase, mitochondrial | 4.43E-10 | 2.39 |
| O95870 | ABHGA | Phosphatidylserine lipase ABHD16A | 1.46E-08 | 2.38 |
| P00439 | PH4H | Phenylalanine-4-hydroxylase | 7.01E-08 | 2.38 |
| Q13464 | ROCK1 | Rho-associated protein kinase 1 | 1.51E-09 | 2.38 |
| Q6UWP7 | LCLT1 | Lysocardiolipin acyltransferase 1 | 1.24E-07 | 2.38 |
| Q6NUS8 | UD3A1 | UDP-glucuronosyltransferase 3A1 | 5.04E-05 | 2.38 |
| Q8IUX4;Q96AK3 | ABC3F;ABC3D | DNA dC->dU-editing enzyme APOBEC-3F;DNA dC->dU-editing enzyme APOBEC-3D | 3.15E-07 | 2.37 |
| P32189 | GLPK | Glycerol kinase | 5.57E-07 | 2.37 |
| P37059 | DHB2 | Estradiol 17-beta-dehydrogenase 2 | 8.79E-08 | 2.37 |
| P26572 | MGAT1 | Alpha-1,3-mannosyl-glycoprotein 2-beta-N-acetylglucosaminyltransferase | 6.88E-08 | 2.37 |
| P07332 | FES | Tyrosine-protein kinase Fes/Fps | 2.11E-06 | 2.37 |
| Q86VD7 | S2542 | Mitochondrial coenzyme A transporter SLC25A42 | 2.00E-08 | 2.36 |
| Q99643 | C560 | Succinate dehydrogenase cytochrome b560 subunit, mitochondrial | 3.02E-07 | 2.36 |
| P41252 | SYIC | Isoleucine--tRNA ligase, cytoplasmic | 2.21E-08 | 2.35 |
| Q13315 | ATM | Serine-protein kinase ATM | 1.05E-05 | 2.34 |
| A0AVT1 | UBA6 | Ubiquitin-like modifier-activating enzyme 6 | 1.03E-10 | 2.32 |
| P61803 | DAD1 | Dolichyl-diphosphooligosaccharide--protein glycosyltransferase subunit DAD1 | 2.15E-08 | 2.31 |
| Q9NUQ7 | UFSP2 | Ufm1-specific protease 2 | 5.04E-09 | 2.31 |
| Q96I59 | SYNM | Probable asparagine--tRNA ligase, mitochondrial | 2.45E-08 | 2.31 |
| Q04446 | GLGB | 1,4-alpha-glucan-branching enzyme | 9.84E-07 | 2.30 |
| Q6PD62 | CTR9 | RNA polymerase-associated protein CTR9 homolog | 5.90E-07 | 2.30 |
| Q13393 | PLD1 | Phospholipase D1 | 2.98E-05 | 2.30 |
| O95671 | ASML | Probable bifunctional dTTP/UTP pyrophosphatase/methyltransferase protein | 3.73E-07 | 2.29 |
| Q13488 | VPP3 | V-type proton ATPase 116 kDa subunit a isoform 3 | 1.98E-07 | 2.29 |
| Q9NRY4 | RHG35 | Rho GTPase-activating protein 35 | 2.03E-11 | 2.29 |
| Q16706 | MA2A1 | Alpha-mannosidase 2 | 3.61E-10 | 2.28 |
| Q9UI12 | VATH | V-type proton ATPase subunit H | 8.16E-10 | 2.27 |
| P00973 | OAS1 | 2'-5'-oligoadenylate synthase 1 | 2.02E-03 | 2.27 |
| Q9H8H3 | MET7A | Methyltransferase-like protein 7A | 1.41E-06 | 2.27 |
| O95340 | PAPS2 | Bifunctional 3'-phosphoadenosine 5'-phosphosulfate synthase 2 | 1.29E-10 | 2.27 |
| P07327 | ADH1A | Alcohol dehydrogenase 1A | 5.48E-11 | 2.26 |
| Q9NZ08 | ERAP1 | Endoplasmic reticulum aminopeptidase 1 | 2.78E-06 | 2.26 |
| Q99460 | PSMD1 | 26S proteasome non-ATPase regulatory subunit 1 | 4.17E-10 | 2.26 |
| P36537 | UDB10 | UDP-glucuronosyltransferase 2B10 | 2.15E-05 | 2.26 |
| Q7Z6Z7 | HUWE1 | E3 ubiquitin-protein ligase HUWE1 | 8.95E-10 | 2.26 |
| Q9Y2L1 | RRP44 | Exosome complex exonuclease RRP44 | 1.19E-07 | 2.25 |
| O75845 | SC5D | Lathosterol oxidase | 1.63E-04 | 2.25 |
| P05165 | PCCA | Propionyl-CoA carboxylase alpha chain, mitochondrial | 1.10E-08 | 2.25 |
| O15084 | ANR28 | Serine/threonine-protein phosphatase 6 regulatory ankyrin repeat subunit A | 4.81E-11 | 2.25 |
| O43813 | LANC1 | Glutathione S-transferase LANCL1 | 1.10E-13 | 2.24 |
| Q99943 | PLCA | 1-acyl-sn-glycerol-3-phosphate acyltransferase alpha | 1.40E-05 | 2.24 |
| Q15125 | EBP | 3-beta-hydroxysteroid-Delta(8),Delta(7)-isomerase | 2.74E-07 | 2.23 |
| Q7L2J0 | MEPCE | 7SK snRNA methylphosphate capping enzyme | 5.12E-03 | 2.23 |
| O95573 | ACSL3 | Long-chain-fatty-acid--CoA ligase 3 | 9.51E-08 | 2.23 |
| Q02318 | CP27A | Sterol 26-hydroxylase, mitochondrial | 9.60E-06 | 2.23 |
| O15111 | IKKA | Inhibitor of nuclear factor kappa-B kinase subunit alpha | 7.49E-08 | 2.22 |
| Q02880 | TOP2B | DNA topoisomerase 2-beta | 1.04E-05 | 2.22 |
| Q9ULT8 | HECD1 | E3 ubiquitin-protein ligase HECTD1 | 1.03E-06 | 2.22 |
| Q86TI2 | DPP9 | Dipeptidyl peptidase 9 | 1.06E-08 | 2.22 |
| Q96NU7 | HUTI | Probable imidazolonepropionase | 3.56E-09 | 2.21 |
| P12235 | ADT1 | ADP/ATP translocase 1 | 4.03E-07 | 2.20 |
| Q13155 | AIMP2 | Aminoacyl tRNA synthase complex-interacting multifunctional protein 2 | 9.86E-11 | 2.20 |
| Q9NRA0 | SPHK2 | Sphingosine kinase 2 | 4.27E-07 | 2.20 |
| P11150 | LIPC | Hepatic triacylglycerol lipase | 6.58E-07 | 2.19 |
| P35558 | PCKGC | Phosphoenolpyruvate carboxykinase, cytosolic [GTP] | 2.78E-06 | 2.18 |
| Q7L5N7 | PCAT2 | Lysophosphatidylcholine acyltransferase 2 | 8.40E-03 | 2.18 |
| Q16719 | KYNU | Kynureninase | 5.06E-07 | 2.18 |
| Q9NR19 | ACSA | Acetyl-coenzyme A synthetase, cytoplasmic | 4.49E-08 | 2.17 |
| O95822 | DCMC | Malonyl-CoA decarboxylase, mitochondrial | 4.94E-07 | 2.16 |
| P08319 | ADH4 | All-trans-retinol dehydrogenase [NAD(+)] ADH4 | 2.39E-06 | 2.16 |
| Q13554 | KCC2B | Calcium/calmodulin-dependent protein kinase type II subunit beta | 4.98E-13 | 2.16 |
| P09960 | LKHA4 | Leukotriene A-4 hydrolase | 4.07E-09 | 2.16 |
| O95954 | FTCD | Formimidoyltransferase-cyclodeaminase | 6.85E-07 | 2.16 |
| P49326 | FMO5 | Dimethylaniline monooxygenase [N-oxide-forming] 5 | 6.10E-08 | 2.15 |
| P17858 | PFKAL | ATP-dependent 6-phosphofructokinase, liver type | 1.10E-08 | 2.15 |
| Q06520 | ST2A1 | Bile salt sulfotransferase | 1.58E-07 | 2.15 |
| Q13268 | DHRS2 | Dehydrogenase/reductase SDR family member 2, mitochondrial | 4.59E-05 | 2.14 |
| Q14C86 | GAPD1 | GTPase-activating protein and VPS9 domain-containing protein 1 | 1.24E-06 | 2.14 |
| P12236 | ADT3 | ADP/ATP translocase 3 | 1.07E-06 | 2.14 |
| Q7KZN9 | COX15 | Cytochrome c oxidase assembly protein COX15 homolog | 6.13E-06 | 2.13 |
| O94966 | UBP19 | Ubiquitin carboxyl-terminal hydrolase 19 | 1.84E-07 | 2.13 |
| P00326 | ADH1G | Alcohol dehydrogenase 1C | 9.87E-13 | 2.13 |
| P30566 | PUR8 | Adenylosuccinate lyase | 7.36E-09 | 2.13 |
| Q9Y5P6 | GMPPB | Mannose-1-phosphate guanyltransferase beta | 2.49E-09 | 2.12 |
| Q8N661 | TM86B | Lysoplasmalogenase | 1.40E-05 | 2.12 |
| Q92835 | SHIP1 | Phosphatidylinositol 3,4,5-trisphosphate 5-phosphatase 1 | 1.18E-04 | 2.11 |
| P00325 | ADH1B | All-trans-retinol dehydrogenase [NAD(+)] ADH1B | 2.58E-11 | 2.11 |
| O15270 | SPTC2 | Serine palmitoyltransferase 2 | 7.96E-07 | 2.11 |
| Q8WVC6 | DCAKD | Dephospho-CoA kinase domain-containing protein | 3.67E-06 | 2.11 |
| P28332 | ADH6 | Alcohol dehydrogenase 6 | 1.01E-09 | 2.11 |
| Q9H993 | ARMT1 | Damage-control phosphatase ARMT1 | 1.41E-07 | 2.11 |
| Q6UWM9 | UD2A3 | UDP-glucuronosyltransferase 2A3 | 2.63E-04 | 2.10 |
| P48729 | KC1A | Casein kinase I isoform alpha | 2.03E-09 | 2.10 |
| Q9UF12 | HYPDH | Hydroxyproline dehydrogenase | 3.64E-06 | 2.10 |
| P0DN79;P35520 | CBSL;CBS | Cystathionine beta-synthase-like protein;Cystathionine beta-synthase | 9.99E-10 | 2.09 |
| Q8NBX0 | SCPDL | Saccharopine dehydrogenase-like oxidoreductase | 2.39E-10 | 2.09 |
| Q5VZE5 | NAA35 | N-alpha-acetyltransferase 35, NatC auxiliary subunit | 6.63E-04 | 2.08 |
| P10620 | MGST1 | Microsomal glutathione S-transferase 1 | 1.60E-07 | 2.08 |
| Q6Y1H2 | HACD2 | Very-long-chain (3R)-3-hydroxyacyl-CoA dehydratase 2 | 9.43E-07 | 2.08 |
| Q9HCG7 | GBA2 | Non-lysosomal glucosylceramidase | 3.43E-05 | 2.08 |
| Q9H6V9 | LDAH | Lipid droplet-associated hydrolase | 7.24E-05 | 2.08 |
| Q01415 | GALK2 | N-acetylgalactosamine kinase | 3.19E-07 | 2.08 |
| P54840 | GYS2 | Glycogen [starch] synthase, liver | 1.10E-05 | 2.07 |
| Q9UNM6 | PSD13 | 26S proteasome non-ATPase regulatory subunit 13 | 3.04E-07 | 2.07 |
| Q8N9L9 | ACOT4 | Peroxisomal succinyl-coenzyme A thioesterase | 3.12E-04 | 2.07 |
| Q93084 | AT2A3 | Sarcoplasmic/endoplasmic reticulum calcium ATPase 3 | 1.98E-04 | 2.07 |
| P20711 | DDC | Aromatic-L-amino-acid decarboxylase | 8.59E-04 | 2.07 |
| Q8IXQ6 | PARP9 | Protein mono-ADP-ribosyltransferase PARP9 | 3.35E-07 | 2.06 |
| Q9Y394 | DHRS7 | Dehydrogenase/reductase SDR family member 7 | 7.61E-07 | 2.06 |
| P23378 | GCSP | Glycine dehydrogenase (decarboxylating), mitochondrial | 1.54E-04 | 2.06 |
| O43776 | SYNC | Asparagine--tRNA ligase, cytoplasmic | 6.83E-14 | 2.06 |
| Q8N0W3 | FCSK | L-fucose kinase | 1.10E-07 | 2.05 |
| Q96G23 | CERS2 | Ceramide synthase 2 | 2.35E-06 | 2.05 |
| Q9UG22 | GIMA2 | GTPase IMAP family member 2 | 1.23E-04 | 2.05 |
| P50336 | PPOX | Protoporphyrinogen oxidase | 7.25E-06 | 2.04 |
| O15091 | MRPP3 | Mitochondrial ribonuclease P catalytic subunit | 5.18E-07 | 2.04 |
| P03891 | NU2M | NADH-ubiquinone oxidoreductase chain 2 | 2.71E-06 | 2.04 |
| P19474 | RO52 | E3 ubiquitin-protein ligase TRIM21 | 4.60E-10 | 2.04 |
| P35575 | G6PC | Glucose-6-phosphatase | 9.10E-07 | 2.04 |
| Q9UPN7 | PP6R1 | Serine/threonine-protein phosphatase 6 regulatory subunit 1 | 7.59E-06 | 2.04 |
| P11216 | PYGB | Glycogen phosphorylase, brain form | 7.37E-07 | 2.04 |
| P49917 | DNLI4 | DNA ligase 4 | 3.78E-05 | 2.03 |
| P52888 | THOP1 | Thimet oligopeptidase | 6.57E-08 | 2.03 |
| Q7L5Y1 | ENOF1 | Mitochondrial enolase superfamily member 1 | 6.73E-08 | 2.03 |
| Q8IU85 | KCC1D | Calcium/calmodulin-dependent protein kinase type 1D | 3.38E-07 | 2.02 |
| P54855 | UDB15 | UDP-glucuronosyltransferase 2B15 | 6.33E-05 | 2.02 |
| P42345 | MTOR | Serine/threonine-protein kinase mTOR | 3.34E-07 | 2.02 |
| P05141 | ADT2 | ADP/ATP translocase 2 | 1.34E-07 | 2.02 |
| Q6NUM9 | RETST | All-trans-retinol 13,14-reductase | 8.89E-06 | 2.02 |
| Q92643 | GPI8 | GPI-anchor transamidase | 1.52E-06 | 2.02 |
| Q9P2J5 | SYLC | Leucine--tRNA ligase, cytoplasmic | 3.47E-09 | 2.01 |
| Q8NFW8 | NEUA | N-acylneuraminate cytidylyltransferase | 2.74E-13 | 2.01 |
| P25098 | ARBK1 | Beta-adrenergic receptor kinase 1 | 5.84E-06 | 2.00 |
| Q10469 | MGAT2 | Alpha-1,6-mannosyl-glycoprotein 2-beta-N-acetylglucosaminyltransferase | 4.20E-05 | 2.00 |
| O43709 | BUD23 | Probable 18S rRNA (guanine-N(7))-methyltransferase | 2.47E-05 | -2.00 |
| O95050 | INMT | Indolethylamine N-methyltransferase | 3.08E-04 | -2.01 |
| Q9P0J7 | KCMF1 | E3 ubiquitin-protein ligase KCMF1 | 1.32E-08 | -2.02 |
| Q7Z4G4 | TRM11 | tRNA (guanine(10)-N2)-methyltransferase homolog | 9.28E-03 | -2.03 |
| Q9BZL4 | PP12C | Protein phosphatase 1 regulatory subunit 12C | 1.32E-16 | -2.04 |
| O00422 | SAP18 | Histone deacetylase complex subunit SAP18 | 5.40E-10 | -2.04 |
| Q16864 | VATF | V-type proton ATPase subunit F | 4.23E-04 | -2.04 |
| P26885 | FKBP2 | Peptidyl-prolyl cis-trans isomerase FKBP2 | 8.28E-10 | -2.05 |
| Q8N7H5 | PAF1 | RNA polymerase II-associated factor 1 homolog | 1.21E-07 | -2.05 |
| P36959 | GMPR1 | GMP reductase 1 | 2.46E-06 | -2.05 |
| Q99807 | COQ7 | 5-demethoxyubiquinone hydroxylase, mitochondrial | 1.37E-07 | -2.07 |
| Q8N0X4 | CLYBL | Citramalyl-CoA lyase, mitochondrial | 5.03E-08 | -2.07 |
| P09622 | DLDH | Dihydrolipoyl dehydrogenase, mitochondrial | 1.28E-10 | -2.07 |
| Q9NPF5 | DMAP1 | DNA methyltransferase 1-associated protein 1 | 1.81E-03 | -2.08 |
| Q9NPJ3 | ACO13 | Acyl-coenzyme A thioesterase 13 | 6.67E-08 | -2.08 |
| Q9Y680 | FKBP7 | Peptidyl-prolyl cis-trans isomerase FKBP7 | 3.00E-10 | -2.08 |
| A0A0B4J2D5;P0DPI2 | GAL3B;GAL3A | Glutamine amidotransferase-like class 1 domain-containing protein 3B, mitochondrial;Glutamine amidotransferase-like class 1 domain-containing protein 3A, mitochondrial | 1.53E-10 | -2.10 |
| O95707 | RPP29 | Ribonuclease P protein subunit p29 | 7.61E-05 | -2.11 |
| P29966 | MARCS | Myristoylated alanine-rich C-kinase substrate | 1.69E-07 | -2.11 |
| P52758 | RIDA | 2-iminobutanoate/2-iminopropanoate deaminase | 7.31E-06 | -2.11 |
| Q8N5L8 | RP25L | Ribonuclease P protein subunit p25-like protein | 6.51E-08 | -2.16 |
| Q712K3 | UB2R2 | Ubiquitin-conjugating enzyme E2 R2 | 2.15E-06 | -2.17 |
| O43914 | TYOBP | TYRO protein tyrosine kinase-binding protein | 9.15E-06 | -2.18 |
| Q9BSE5 | SPEB | Agmatinase, mitochondrial | 1.96E-05 | -2.20 |
| P63146 | UBE2B | Ubiquitin-conjugating enzyme E2 B | 1.04E-10 | -2.20 |
| O75947 | ATP5H | ATP synthase subunit d, mitochondrial | 1.30E-09 | -2.20 |
| P54710 | ATNG | Sodium/potassium-transporting ATPase subunit gamma | 6.38E-03 | -2.23 |
| P32320 | CDD | Cytidine deaminase | 1.74E-06 | -2.26 |
| Q96A00 | PP14A | Protein phosphatase 1 regulatory subunit 14A | 2.09E-06 | -2.26 |
| Q96B97 | SH3K1 | SH3 domain-containing kinase-binding protein 1 | 1.95E-03 | -2.27 |
| P47895 | AL1A3 | Aldehyde dehydrogenase family 1 member A3 | 6.77E-07 | -2.29 |
| P51970 | NDUA8 | NADH dehydrogenase [ubiquinone] 1 alpha subcomplex subunit 8 | 3.93E-09 | -2.31 |
| P14621 | ACYP2 | Acylphosphatase-2 | 2.36E-05 | -2.31 |
| Q16775 | GLO2 | Hydroxyacylglutathione hydrolase, mitochondrial | 4.79E-08 | -2.32 |
| Q8NFU3 | TSTD1 | Thiosulfate:glutathione sulfurtransferase | 4.50E-06 | -2.32 |
| Q9Y508 | RN114 | E3 ubiquitin-protein ligase RNF114 | 5.82E-09 | -2.32 |
| P10586 | PTPRF | Receptor-type tyrosine-protein phosphatase F | 8.72E-11 | -2.33 |
| Q9UN86 | G3BP2 | Ras GTPase-activating protein-binding protein 2 | 2.32E-11 | -2.37 |
| P20933 | ASPG | N(4)-(beta-N-acetylglucosaminyl)-L-asparaginase | 6.04E-07 | -2.38 |
| O95182 | NDUA7 | NADH dehydrogenase [ubiquinone] 1 alpha subcomplex subunit 7 | 3.55E-07 | -2.38 |
| Q00688 | FKBP3 | Peptidyl-prolyl cis-trans isomerase FKBP3 | 3.27E-11 | -2.39 |
| Q17R31 | TATD3 | Putative deoxyribonuclease TATDN3 | 2.24E-07 | -2.39 |
| Q9H0N5 | PHS2 | Pterin-4-alpha-carbinolamine dehydratase 2 | 2.26E-05 | -2.40 |
| P45877 | PPIC | Peptidyl-prolyl cis-trans isomerase C | 2.53E-07 | -2.40 |
| P60484 | PTEN | Phosphatidylinositol 3,4,5-trisphosphate 3-phosphatase and dual-specificity protein phosphatase PTEN | 2.87E-03 | -2.44 |
| Q99497 | PARK7 | Protein/nucleic acid deglycase DJ-1 | 4.90E-08 | -2.45 |
| P15169 | CBPN | Carboxypeptidase N catalytic chain | 2.09E-07 | -2.45 |
| P53041 | PPP5 | Serine/threonine-protein phosphatase 5 | 9.72E-05 | -2.46 |
| P15954 | COX7C | Cytochrome c oxidase subunit 7C, mitochondrial | 1.26E-03 | -2.47 |
| P04745 | AMY1 | Alpha-amylase 1 | 7.45E-07 | -2.47 |
| Q96C90 | PP14B | Protein phosphatase 1 regulatory subunit 14B | 5.29E-10 | -2.49 |
| Q9UII2 | ATIF1 | ATPase inhibitor, mitochondrial | 3.15E-05 | -2.49 |
| P30049 | ATPD | ATP synthase subunit delta, mitochondrial | 6.11E-09 | -2.49 |
| P20674 | COX5A | Cytochrome c oxidase subunit 5A, mitochondrial | 1.09E-07 | -2.53 |
| Q9C0C2 | TB182 | 182 kDa tankyrase-1-binding protein | 1.13E-09 | -2.54 |
| Q13526 | PIN1 | Peptidyl-prolyl cis-trans isomerase NIMA-interacting 1 | 3.53E-07 | -2.54 |
| O60220 | TIM8A | Mitochondrial import inner membrane translocase subunit Tim8 A | 2.74E-07 | -2.55 |
| P33316 | DUT | Deoxyuridine 5'-triphosphate nucleotidohydrolase, mitochondrial | 6.77E-11 | -2.56 |
| Q9Y5J6 | T10B | Mitochondrial import inner membrane translocase subunit Tim10 B | 3.11E-09 | -2.56 |
| O60237 | MYPT2 | Protein phosphatase 1 regulatory subunit 12B | 5.36E-08 | -2.59 |
| Q93091 | RNAS6 | Ribonuclease K6 | 5.06E-08 | -2.60 |
| O75380 | NDUS6 | NADH dehydrogenase [ubiquinone] iron-sulfur protein 6, mitochondrial | 8.32E-04 | -2.61 |
| Q9NSY1 | BMP2K | BMP-2-inducible protein kinase | 4.61E-08 | -2.66 |
| O00233 | PSMD9 | 26S proteasome non-ATPase regulatory subunit 9 | 3.98E-08 | -2.67 |
| O43865 | SAHH2 | S-adenosylhomocysteine hydrolase-like protein 1 | 7.92E-03 | -2.68 |
| P30046 | DOPD | D-dopachrome decarboxylase | 2.88E-03 | -2.69 |
| P46527 | CDN1B | Cyclin-dependent kinase inhibitor 1B | 4.97E-05 | -2.74 |
| Q96BR5 | COA7 | Cytochrome c oxidase assembly factor 7 | 1.28E-10 | -2.79 |
| P56181 | NDUV3 | NADH dehydrogenase [ubiquinone] flavoprotein 3, mitochondrial | 2.60E-05 | -2.85 |
| Q96PE7 | MCEE | Methylmalonyl-CoA epimerase, mitochondrial | 9.06E-10 | -2.87 |
| O43678 | NDUA2 | NADH dehydrogenase [ubiquinone] 1 alpha subcomplex subunit 2 | 1.92E-07 | -2.89 |
| O43716 | GATC | Glutamyl-tRNA(Gln) amidotransferase subunit C, mitochondrial | 1.20E-08 | -2.99 |
| P53999 | TCP4 | Activated RNA polymerase II transcriptional coactivator p15 | 2.01E-10 | -3.03 |
| Q9H1K1 | ISCU | Iron-sulfur cluster assembly enzyme ISCU, mitochondrial | 2.72E-04 | -3.06 |
| Q13283 | G3BP1 | Ras GTPase-activating protein-binding protein 1 | 4.59E-12 | -3.08 |
| Q9Y237 | PIN4 | Peptidyl-prolyl cis-trans isomerase NIMA-interacting 4 | 6.02E-07 | -3.08 |
| P04180 | LCAT | Phosphatidylcholine-sterol acyltransferase | 9.56E-06 | -3.14 |
| Q9BQ61 | TRIR | Telomerase RNA component interacting RNase | 1.82E-07 | -3.19 |
| P22352 | GPX3 | Glutathione peroxidase 3 | 4.19E-10 | -3.19 |
| Q9BX93 | PG12B | Group XIIB secretory phospholipase A2-like protein | 6.28E-09 | -3.25 |
| Q9BY77 | PDIP3 | Polymerase delta-interacting protein 3 | 3.58E-08 | -3.26 |
| P14854 | CX6B1 | Cytochrome c oxidase subunit 6B1 | 4.50E-10 | -3.36 |
| O14519;O75956 | CDKA1;CDKA2 | Cyclin-dependent kinase 2-associated protein 1;Cyclin-dependent kinase 2-associated protein 2 | 5.95E-10 | -3.39 |
| O43181 | NDUS4 | NADH dehydrogenase [ubiquinone] iron-sulfur protein 4, mitochondrial | 2.64E-10 | -3.40 |
| Q9NRX4 | PHP14 | 14 kDa phosphohistidine phosphatase | 1.15E-10 | -3.41 |
| Q8NBP7 | PCSK9 | Proprotein convertase subtilisin/kexin type 9 | 4.29E-17 | -3.44 |
| P10153 | RNAS2 | Non-secretory ribonuclease | 1.60E-08 | -3.45 |
| Q9Y5J7 | TIM9 | Mitochondrial import inner membrane translocase subunit Tim9 | 3.96E-08 | -3.47 |
| P62942 | FKB1A | Peptidyl-prolyl cis-trans isomerase FKBP1A | 3.69E-09 | -3.48 |
| P08294 | SODE | Extracellular superoxide dismutase [Cu-Zn] | 4.35E-11 | -3.49 |
| Q9H1E3 | NUCKS | Nuclear ubiquitous casein and cyclin-dependent kinase substrate 1 | 2.80E-06 | -3.52 |
| Q9NPI6 | DCP1A | mRNA-decapping enzyme 1A | 5.12E-06 | -3.54 |
| Q03013 | GSTM4 | Glutathione S-transferase Mu 4 | 1.43E-03 | -3.67 |
| Q8IZ21 | PHAR4 | Phosphatase and actin regulator 4 | 1.84E-09 | -3.81 |
| A1L188 | NDUF8 | NADH dehydrogenase [ubiquinone] 1 alpha subcomplex assembly factor 8 | 6.76E-09 | -3.95 |
| P15085 | CBPA1 | Carboxypeptidase A1 | 5.03E-05 | -3.99 |
| P30405 | PPIF | Peptidyl-prolyl cis-trans isomerase F, mitochondrial | 6.17E-13 | -4.06 |
| P49841 | GSK3B | Glycogen synthase kinase-3 beta | 1.07E-05 | -4.11 |
| Q8IWW6 | RHG12 | Rho GTPase-activating protein 12 | 8.13E-11 | -4.11 |
| P00441 | SODC | Superoxide dismutase [Cu-Zn] | 3.20E-06 | -4.14 |
| Q9BXJ9 | NAA15 | N-alpha-acetyltransferase 15, NatA auxiliary subunit | 1.95E-06 | -4.23 |
| Q5JTJ3 | COA6 | Cytochrome c oxidase assembly factor 6 homolog | 4.65E-09 | -4.25 |
| P18859 | ATP5J | ATP synthase-coupling factor 6, mitochondrial | 3.59E-07 | -4.35 |
| Q7Z2Z2 | EFL1 | Elongation factor-like GTPase 1 | 3.13E-08 | -5.07 |
| Q9Y3D2 | MSRB2 | Methionine-R-sulfoxide reductase B2, mitochondrial | 3.84E-08 | -5.12 |
| Q86WW8 | COA5 | Cytochrome c oxidase assembly factor 5 | 2.08E-04 | -5.83 |
| P23280 | CAH6 | Carbonic anhydrase 6 | 2.23E-04 | -5.86 |
| Q9Y5J9 | TIM8B | Mitochondrial import inner membrane translocase subunit Tim8 B | 1.70E-10 | -5.90 |
| P09093 | CEL3A | Chymotrypsin-like elastase family member 3A | 5.79E-04 | -6.09 |
| P10606 | COX5B | Cytochrome c oxidase subunit 5B, mitochondrial | 3.85E-07 | -6.81 |
| Q9Y5L4 | TIM13 | Mitochondrial import inner membrane translocase subunit Tim13 | 1.54E-07 | -7.27 |
| Q9C0H9 | SRCN1 | SRC kinase signaling inhibitor 1 | 3.98E-04 | -8.13 |
| P06732 | KCRM | Creatine kinase M-type | 1.25E-15 | -8.55 |
| Q6ZXV5 | TMTC3 | Protein O-mannosyl-transferase TMTC3 | 2.10E-07 | -13.00 |
| P07205 | PGK2 | Phosphoglycerate kinase 2 | 4.51E-03 | -14.66 |

*P* value < 0.01, |fold change| > 2.

**Table S7. Enzymes and enzyme-related proteins in differential proteins in the IFLT_PR versus CLT_PR group.**

| UniProt ID | Protein Name | Protein Description | *P* Value | Fold Change |
| --- | --- | --- | --- | --- |
| Q5T447 | HECD3 | E3 ubiquitin-protein ligase HECTD3 | 1.05E-12 | 7.44 |
| Q3KRA9 | ALKB6 | Alpha-ketoglutarate-dependent dioxygenase alkB homolog 6 | 1.85E-05 | 5.83 |
| O00204 | ST2B1 | Sulfotransferase 2B1 | 1.05E-03 | 4.03 |
| P50993 | AT1A2 | Sodium/potassium-transporting ATPase subunit alpha-2 | 5.90E-06 | 3.33 |
| P04746 | AMYP | Pancreatic alpha-amylase | 8.12E-03 | 3.22 |
| Q9HBK9 | AS3MT | Arsenite methyltransferase | 3.52E-08 | 2.90 |
| O75936 | BODG | Gamma-butyrobetaine dioxygenase | 2.30E-05 | 2.83 |
| P42357 | HUTH | Histidine ammonia-lyase | 8.24E-11 | 2.73 |
| P30711 | GSTT1 | Glutathione S-transferase theta-1 | 7.18E-05 | 2.55 |
| P35573 | GDE | Glycogen debranching enzyme | 1.09E-11 | 2.55 |
| O60218 | AK1BA | Aldo-keto reductase family 1 member B10 | 1.04E-04 | 2.51 |
| Q6P6C2 | ALKB5 | RNA demethylase ALKBH5 | 8.72E-05 | 2.46 |
| P13196 | HEM1 | 5-aminolevulinate synthase, nonspecific, mitochondrial | 3.34E-04 | 2.46 |
| Q12882 | DPYD | Dihydropyrimidine dehydrogenase [NADP(+)] | 1.02E-13 | 2.40 |
| Q8IV48 | ERI1 | 3'-5' exoribonuclease 1 | 7.62E-05 | 2.36 |
| Q14397 | GCKR | Glucokinase regulatory protein | 9.25E-08 | 2.35 |
| Q5TFE4 | NT5D1 | 5'-nucleotidase domain-containing protein 1 | 1.00E-12 | 2.28 |
| P07327 | ADH1A | Alcohol dehydrogenase 1A | 8.15E-10 | 2.26 |
| P28332 | ADH6 | Alcohol dehydrogenase 6 | 2.16E-09 | 2.19 |
| Q93099 | HGD | Homogentisate 1,2-dioxygenase | 1.20E-10 | 2.17 |
| P17516 | AK1C4 | Aldo-keto reductase family 1 member C4 | 6.04E-11 | 2.12 |
| Q14914 | PTGR1 | Prostaglandin reductase 1 | 3.68E-10 | 2.12 |
| P20132 | SDHL | L-serine dehydratase/L-threonine deaminase | 5.01E-04 | 2.10 |
| P15088 | CBPA3 | Mast cell carboxypeptidase A | 1.26E-07 | 2.10 |
| Q8N6H7 | ARFG2 | ADP-ribosylation factor GTPase-activating protein 2 | 6.96E-11 | 2.10 |
| Q9NYL5 | CP39A | 24-hydroxycholesterol 7-alpha-hydroxylase | 7.51E-04 | 2.10 |
| P00326 | ADH1G | Alcohol dehydrogenase 1C | 1.31E-09 | 2.07 |
| Q9BVL4 | SELO | Protein adenylyltransferase SelO, mitochondrial | 1.47E-11 | 2.07 |
| Q9H4B0 | OSGP2 | Probable tRNA N6-adenosine threonylcarbamoyltransferase, mitochondrial | 9.35E-06 | 2.06 |
| Q9BW92 | SYTM | Threonine--tRNA ligase, mitochondrial | 3.04E-15 | 2.02 |
| Q7L2E3 | DHX30 | ATP-dependent RNA helicase DHX30 | 7.34E-08 | 2.02 |
| Q96F10 | SAT2 | Diamine acetyltransferase 2 | 3.36E-09 | 2.01 |
| O75884 | RBBP9 | Serine hydrolase RBBP9 | 5.86E-12 | 2.01 |
| O95376 | ARI2 | E3 ubiquitin-protein ligase ARIH2 | 4.24E-03 | 2.00 |
| P13284 | GILT | Gamma-interferon-inducible lysosomal thiol reductase | 3.45E-06 | -2.03 |
| Q96IY4 | CBPB2 | Carboxypeptidase B2 | 2.47E-05 | -2.04 |
| O75563 | SKAP2 | Src kinase-associated phosphoprotein 2 | 1.05E-10 | -2.04 |
| P06276 | CHLE | Cholinesterase | 1.38E-03 | -2.04 |
| Q96DA6 | TIM14 | Mitochondrial import inner membrane translocase subunit TIM14 | 1.68E-07 | -2.04 |
| P11413 | G6PD | Glucose-6-phosphate 1-dehydrogenase | 3.00E-03 | -2.07 |
| Q9H0N5 | PHS2 | Pterin-4-alpha-carbinolamine dehydratase 2 | 1.06E-04 | -2.07 |
| Q5MY95 | ENTP8 | Ectonucleoside triphosphate diphosphohydrolase 8 | 3.31E-02 | -2.08 |
| P48052 | CBPA2 | Carboxypeptidase A2 | 3.66E-05 | -2.08 |
| Q68CQ7 | GL8D1 | Glycosyltransferase 8 domain-containing protein 1 | 3.13E-05 | -2.08 |
| Q70EL4 | UBP43 | Ubiquitin carboxyl-terminal hydrolase 43 | 4.64E-02 | -2.09 |
| P35575 | G6PC | Glucose-6-phosphatase | 2.64E-02 | -2.10 |
| P45877 | PPIC | Peptidyl-prolyl cis-trans isomerase C | 1.92E-03 | -2.10 |
| Q9UN86 | G3BP2 | Ras GTPase-activating protein-binding protein 2 | 1.71E-05 | -2.10 |
| P53999 | TCP4 | Activated RNA polymerase II transcriptional coactivator p15 | 2.79E-05 | -2.11 |
| P08397 | HEM3 | Porphobilinogen deaminase | 1.53E-10 | -2.12 |
| Q8N4C8 | MINK1 | Misshapen-like kinase 1 | 1.05E-03 | -2.12 |
| O94822 | LTN1 | E3 ubiquitin-protein ligase listerin | 1.98E-06 | -2.12 |
| P20933 | ASPG | N(4)-(beta-N-acetylglucosaminyl)-L-asparaginase | 4.48E-07 | -2.12 |
| Q9Y680 | FKBP7 | Peptidyl-prolyl cis-trans isomerase FKBP7 | 1.59E-04 | -2.12 |
| Q9BX93 | PG12B | Group XIIB secretory phospholipase A2-like protein | 2.60E-07 | -2.12 |
| Q9H1K1 | ISCU | Iron-sulfur cluster assembly enzyme ISCU, mitochondrial | 2.11E-03 | -2.13 |
| Q9NRF9 | DPOE3 | DNA polymerase epsilon subunit 3 | 4.43E-05 | -2.13 |
| Q99595 | TI17A | Mitochondrial import inner membrane translocase subunit Tim17-A | 7.25E-07 | -2.15 |
| P09172 | DOPO | Dopamine beta-hydroxylase | 2.75E-02 | -2.15 |
| Q16864 | VATF | V-type proton ATPase subunit F | 6.06E-06 | -2.17 |
| P29966 | MARCS | Myristoylated alanine-rich C-kinase substrate | 1.25E-08 | -2.18 |
| Q8NHG8 | ZNRF2 | E3 ubiquitin-protein ligase ZNRF2 | 3.94E-05 | -2.19 |
| Q8WZ82 | OVCA2 | Esterase OVCA2 | 2.34E-02 | -2.20 |
| Q8NBP7 | PCSK9 | Proprotein convertase subtilisin/kexin type 9 | 5.68E-04 | -2.20 |
| Q99497 | PARK7 | Protein/nucleic acid deglycase DJ-1 | 4.71E-07 | -2.21 |
| Q9NSY1 | BMP2K | BMP-2-inducible protein kinase | 4.62E-07 | -2.21 |
| Q6PCE3 | PGM2L | Glucose 1,6-bisphosphate synthase | 1.71E-02 | -2.22 |
| Q7Z6I6 | RHG30 | Rho GTPase-activating protein 30 | 6.48E-08 | -2.22 |
| Q10588 | BST1 | ADP-ribosyl cyclase/cyclic ADP-ribose hydrolase 2 | 2.44E-05 | -2.22 |
| P14780 | MMP9 | Matrix metalloproteinase-9 | 2.00E-03 | -2.22 |
| Q96C90 | PP14B | Protein phosphatase 1 regulatory subunit 14B | 2.22E-04 | -2.22 |
| P12532;P17540 | KCRU;KCRS | Creatine kinase U-type, mitochondrial;Creatine kinase S-type, mitochondrial | 3.16E-04 | -2.23 |
| Q8N1Q1 | CAH13 | Carbonic anhydrase 13 | 1.46E-03 | -2.24 |
| O95182 | NDUA7 | NADH dehydrogenase [ubiquinone] 1 alpha subcomplex subunit 7 | 6.71E-06 | -2.26 |
| Q9NVH0 | EXD2 | Exonuclease 3'-5' domain-containing protein 2 | 1.45E-02 | -2.28 |
| Q12913 | PTPRJ | Receptor-type tyrosine-protein phosphatase eta | 4.17E-03 | -2.29 |
| Q6WCQ1 | MPRIP | Myosin phosphatase Rho-interacting protein | 3.78E-02 | -2.30 |
| Q9BZL4 | PP12C | Protein phosphatase 1 regulatory subunit 12C | 1.09E-05 | -2.31 |
| P36959 | GMPR1 | GMP reductase 1 | 4.65E-07 | -2.32 |
| P01033 | TIMP1 | Metalloproteinase inhibitor 1 | 2.25E-03 | -2.32 |
| P78324 | SHPS1 | Tyrosine-protein phosphatase non-receptor type substrate 1 | 9.77E-12 | -2.33 |
| P20674 | COX5A | Cytochrome c oxidase subunit 5A, mitochondrial | 8.29E-06 | -2.33 |
| P14555 | PA2GA | Phospholipase A2, membrane associated | 1.09E-04 | -2.34 |
| P09488 | GSTM1 | Glutathione S-transferase Mu 1 | 3.00E-03 | -2.34 |
| Q93084 | AT2A3 | Sarcoplasmic/endoplasmic reticulum calcium ATPase 3 | 2.71E-05 | -2.34 |
| P46087 | NOP2 | Probable 28S rRNA (cytosine(4447)-C(5))-methyltransferase | 2.55E-02 | -2.37 |
| O15460 | P4HA2 | Prolyl 4-hydroxylase subunit alpha-2 | 1.28E-06 | -2.37 |
| P30043 | BLVRB | Flavin reductase (NADPH) | 2.09E-13 | -2.40 |
| P62487 | RPB7 | DNA-directed RNA polymerase II subunit RPB7 | 5.79E-05 | -2.42 |
| Q93091 | RNAS6 | Ribonuclease K6 | 4.78E-06 | -2.43 |
| O43709 | BUD23 | Probable 18S rRNA (guanine-N(7))-methyltransferase | 1.23E-02 | -2.46 |
| Q9BQ61 | TRIR | Telomerase RNA component interacting RNase | 5.06E-05 | -2.49 |
| P24311 | COX7B | Cytochrome c oxidase subunit 7B, mitochondrial | 2.36E-08 | -2.53 |
| P22792 | CPN2 | Carboxypeptidase N subunit 2 | 1.25E-06 | -2.53 |
| Q6P4A8 | PLBL1 | Phospholipase B-like 1 | 1.20E-05 | -2.53 |
| Q8WTS1 | ABHD5 | 1-acylglycerol-3-phosphate O-acyltransferase ABHD5 | 6.79E-04 | -2.54 |
| P15169 | CBPN | Carboxypeptidase N catalytic chain | 6.98E-04 | -2.56 |
| P14854 | CX6B1 | Cytochrome c oxidase subunit 6B1 | 3.69E-06 | -2.61 |
| Q9UK55 | ZPI | Protein Z-dependent protease inhibitor | 2.27E-05 | -2.65 |
| Q92835 | SHIP1 | Phosphatidylinositol 3,4,5-trisphosphate 5-phosphatase 1 | 3.33E-04 | -2.66 |
| P41247 | PLPL4 | Patatin-like phospholipase domain-containing protein 4 | 1.44E-02 | -2.67 |
| O95352 | ATG7 | Ubiquitin-like modifier-activating enzyme ATG7 | 3.54E-02 | -2.67 |
| P22894 | MMP8 | Neutrophil collagenase | 3.66E-07 | -2.69 |
| Q5VVQ6 | OTU1 | Ubiquitin thioesterase OTU1 | 8.69E-07 | -2.69 |
| Q93070 | NAR4 | Ecto-ADP-ribosyltransferase 4 | 1.16E-04 | -2.69 |
| P15954 | COX7C | Cytochrome c oxidase subunit 7C, mitochondrial | 1.89E-07 | -2.70 |
| Q9C0C2 | TB182 | 182 kDa tankyrase-1-binding protein | 1.75E-06 | -2.71 |
| P08294 | SODE | Extracellular superoxide dismutase [Cu-Zn] | 1.01E-07 | -2.72 |
| P10586 | PTPRF | Receptor-type tyrosine-protein phosphatase F | 1.66E-09 | -2.73 |
| O43181 | NDUS4 | NADH dehydrogenase [ubiquinone] iron-sulfur protein 4, mitochondrial | 7.15E-08 | -2.76 |
| P43378 | PTN9 | Tyrosine-protein phosphatase non-receptor type 9 | 6.53E-03 | -2.79 |
| Q9Y3D7 | TIM16 | Mitochondrial import inner membrane translocase subunit TIM16 | 6.92E-05 | -2.80 |
| O00233 | PSMD9 | 26S proteasome non-ATPase regulatory subunit 9 | 8.76E-10 | -2.92 |
| A1L188 | NDUF8 | NADH dehydrogenase [ubiquinone] 1 alpha subcomplex assembly factor 8 | 6.12E-07 | -2.93 |
| Q9H777 | RNZ1 | Zinc phosphodiesterase ELAC protein 1 | 1.65E-02 | -2.93 |
| P52435;Q9GZM3;Q9H1A7 | RPB11;RPB1B;RPB1C | DNA-directed RNA polymerase II subunit RPB11-a;DNA-directed RNA polymerase II subunit RPB11-b1;DNA-directed RNA polymerase II subunit RPB11-b2 | 2.59E-06 | -2.94 |
| P62942 | FKB1A | Peptidyl-prolyl cis-trans isomerase FKBP1A | 1.47E-08 | -2.96 |
| P49247 | RPIA | Ribose-5-phosphate isomerase | 4.38E-09 | -2.96 |
| Q9Y237 | PIN4 | Peptidyl-prolyl cis-trans isomerase NIMA-interacting 4 | 8.24E-05 | -2.96 |
| P16444 | DPEP1 | Dipeptidase 1 | 1.31E-07 | -3.00 |
| O43716 | GATC | Glutamyl-tRNA(Gln) amidotransferase subunit C, mitochondrial | 3.43E-05 | -3.02 |
| Q96T66 | NMNA3 | Nicotinamide/nicotinic acid mononucleotide adenylyltransferase 3 | 2.29E-03 | -3.03 |
| P11245 | ARY2 | Arylamine N-acetyltransferase 2 | 3.39E-06 | -3.04 |
| Q9BY77 | PDIP3 | Polymerase delta-interacting protein 3 | 7.92E-05 | -3.08 |
| P32321 | DCTD | Deoxycytidylate deaminase | 1.71E-04 | -3.08 |
| Q8NBM8 | PCYXL | Prenylcysteine oxidase-like | 1.65E-02 | -3.23 |
| O00391 | QSOX1 | Sulfhydryl oxidase 1 | 2.45E-05 | -3.25 |
| Q7Z4G4 | TRM11 | tRNA (guanine(10)-N2)-methyltransferase homolog | 8.14E-07 | -3.31 |
| Q9UM07 | PADI4 | Protein-arginine deiminase type-4 | 5.56E-10 | -3.37 |
| Q9BYK8 | HELZ2 | Helicase with zinc finger domain 2 | 2.15E-05 | -3.38 |
| P30405 | PPIF | Peptidyl-prolyl cis-trans isomerase F, mitochondrial | 5.88E-11 | -3.39 |
| O60220 | TIM8A | Mitochondrial import inner membrane translocase subunit Tim8 A | 1.06E-04 | -3.43 |
| P05164 | PERM | Myeloperoxidase | 1.17E-06 | -3.45 |
| P56181 | NDUV3 | NADH dehydrogenase [ubiquinone] flavoprotein 3, mitochondrial | 1.48E-06 | -3.56 |
| P04035 | HMDH | 3-hydroxy-3-methylglutaryl-coenzyme A reductase | 3.40E-04 | -3.56 |
| Q86WW8 | COA5 | Cytochrome c oxidase assembly factor 5 | 4.21E-07 | -3.58 |
| Q96PE7 | MCEE | Methylmalonyl-CoA epimerase, mitochondrial | 3.18E-09 | -3.63 |
| P00441 | SODC | Superoxide dismutase [Cu-Zn] | 1.54E-04 | -3.66 |
| Q6UWY2 | PRS57 | Serine protease 57 | 8.48E-03 | -3.74 |
| Q9H1E3 | NUCKS | Nuclear ubiquitous casein and cyclin-dependent kinase substrate 1 | 1.74E-06 | -3.80 |
| P51512 | MMP16 | Matrix metalloproteinase-16 | 2.34E-06 | -3.82 |
| O60237 | MYPT2 | Protein phosphatase 1 regulatory subunit 12B | 2.49E-08 | -3.87 |
| O43451 | MGA | Maltase-glucoamylase, intestinal | 1.15E-06 | -3.87 |
| Q9Y2Q0 | AT8A1 | Phospholipid-transporting ATPase IA | 2.73E-03 | -3.91 |
| Q8IYD1 | ERF3B | Eukaryotic peptide chain release factor GTP-binding subunit ERF3B | 8.32E-10 | -3.96 |
| O00507 | USP9Y | Probable ubiquitin carboxyl-terminal hydrolase FAF-Y | 7.72E-03 | -3.98 |
| P54710 | ATNG | Sodium/potassium-transporting ATPase subunit gamma | 1.73E-07 | -4.00 |
| P22352 | GPX3 | Glutathione peroxidase 3 | 6.73E-14 | -4.00 |
| P32320 | CDD | Cytidine deaminase | 5.50E-11 | -4.10 |
| O75380 | NDUS6 | NADH dehydrogenase [ubiquinone] iron-sulfur protein 6, mitochondrial | 1.27E-05 | -4.25 |
| P18859 | ATP5J | ATP synthase-coupling factor 6, mitochondrial | 6.47E-05 | -4.28 |
| P49441 | INPP | Inositol polyphosphate 1-phosphatase | 2.44E-04 | -4.31 |
| P80188 | NGAL | Neutrophil gelatinase-associated lipocalin | 6.05E-06 | -4.45 |
| P08246 | ELNE | Neutrophil elastase | 8.39E-05 | -4.45 |
| Q9Y2J8 | PADI2 | Protein-arginine deiminase type-2 | 2.62E-05 | -4.75 |
| Q9NRX4 | PHP14 | 14 kDa phosphohistidine phosphatase | 7.36E-11 | -4.75 |
| Q03013 | GSTM4 | Glutathione S-transferase Mu 4 | 7.15E-04 | -4.79 |
| Q9Y3D2 | MSRB2 | Methionine-R-sulfoxide reductase B2, mitochondrial | 4.58E-04 | -4.82 |
| O43865 | SAHH2 | S-adenosylhomocysteine hydrolase-like protein 1 | 6.87E-09 | -4.82 |
| Q16769 | QPCT | Glutaminyl-peptide cyclotransferase | 9.32E-09 | -4.90 |
| P78563 | RED1 | Double-stranded RNA-specific editase 1 | 2.21E-07 | -4.93 |
| Q13231 | CHIT1 | Chitotriosidase-1 | 3.03E-04 | -5.17 |
| P07451 | CAH3 | Carbonic anhydrase 3 | 5.67E-09 | -5.22 |
| P09769 | FGR | Tyrosine-protein kinase Fgr | 1.92E-09 | -5.26 |
| O15357 | SHIP2 | Phosphatidylinositol 3,4,5-trisphosphate 5-phosphatase 2 | 2.44E-03 | -5.55 |
| Q5JTJ3 | COA6 | Cytochrome c oxidase assembly factor 6 homolog | 1.47E-07 | -5.64 |
| Q7Z2Z2 | EFL1 | Elongation factor-like GTPase 1 | 5.05E-10 | -5.89 |
| P10606 | COX5B | Cytochrome c oxidase subunit 5B, mitochondrial | 5.66E-07 | -6.10 |
| P08861 | CEL3B | Chymotrypsin-like elastase family member 3B | 4.55E-02 | -6.23 |
| Q9Y5L4 | TIM13 | Mitochondrial import inner membrane translocase subunit Tim13 | 9.28E-07 | -6.81 |
| P07738 | PMGE | Bisphosphoglycerate mutase | 5.00E-08 | -6.82 |
| P12931 | SRC | Proto-oncogene tyrosine-protein kinase Src | 6.50E-03 | -7.03 |
| P15086 | CBPB1 | Carboxypeptidase B | 2.42E-02 | -7.98 |
| P00915 | CAH1 | Carbonic anhydrase 1 | 4.16E-09 | -8.41 |
| P10153 | RNAS2 | Non-secretory ribonuclease | 1.07E-11 | -8.86 |
| Q9NR20 | DYRK4 | Dual specificity tyrosine-phosphorylation-regulated kinase 4 | 6.07E-08 | -9.38 |
| P15085 | CBPA1 | Carboxypeptidase A1 | 2.49E-02 | -10.58 |

*P* value < 0.01, |fold change| > 2.
